# Unbiased Simulations Reveal the Inward-Facing Conformation of the Human Serotonin Transporter and Na^+^ Ion Release

**DOI:** 10.1371/journal.pcbi.1002246

**Published:** 2011-10-27

**Authors:** Heidi Koldsø, Pernille Noer, Julie Grouleff, Henriette Elisabeth Autzen, Steffen Sinning, Birgit Schiøtt

**Affiliations:** 1 Center for Insoluble Protein Structures (inSPIN) and Interdisciplinary Nanoscience Center (iNANO), Department of Chemistry, Aarhus University, Aarhus, Denmark; 2 Laboratory of Molecular Neurobiology, Centre for Psychiatric Research, Aarhus University Hospital, Risskov, Denmark; UNC Charlotte, United States of America

## Abstract

Monoamine transporters are responsible for termination of synaptic signaling and are involved in depression, control of appetite, and anxiety amongst other neurological processes. Despite extensive efforts, the structures of the monoamine transporters and the transport mechanism of ions and substrates are still largely unknown. Structural knowledge of the human serotonin transporter (hSERT) is much awaited for understanding the mechanistic details of substrate translocation and binding of antidepressants and drugs of abuse. The publication of the crystal structure of the homologous leucine transporter has resulted in homology models of the monoamine transporters. Here we present extended molecular dynamics simulations of an experimentally supported homology model of hSERT with and without the natural substrate yielding a total of more than 1.5 µs of simulation of the protein dimer. The simulations reveal a transition of hSERT from an outward-facing occluded conformation to an inward-facing conformation in a one-substrate-bound state. Simulations with a second substrate in the proposed symport effector site did not lead to conformational changes associated with translocation. The central substrate binding site becomes fully exposed to the cytoplasm leaving both the Na^+^-ion in the Na2-site and the substrate in direct contact with the cytoplasm through water interactions. The simulations reveal how sodium is released and show indications of early events of substrate transport. The notion that ion dissociation from the Na2-site drives translocation is supported by experimental studies of a Na2-site mutant. Transmembrane helices (TMs) 1 and 6 are identified as the helices involved in the largest movements during transport.

## Author Summary

The human serotonin transporter belongs to the family of neurotransmitter transporters, which are located in the presynaptic nerve end, from where it is responsible for termination of synaptic serotonin signaling. Imbalance in serotonin concentration is related to various neuronal conditions such as depression, regulation of appetite etc. Very limited structural information of hSERT is available, but it is believed that the protein functions through an alternating access mechanism, where the central binding site is either exposed to the outside or the inside of the cell. We have previously published an experimentally validated outward-occluded homology model of hSERT, and here we reveal the inward-facing conformation of hSERT from molecular dynamics simulations, from which we can identify the main movements occurring during the translocation. From the inward-facing conformation we observe ion release, revealing important information on the sequence of events during transport. Following transport of the sodium ion, the substrate also shows early events of transport. The ion follows a cytoplasmic pathway as hinted at from experiments, and the ligand binding site becomes fully solvated by water through this same pathway. Experiments using an Asp437Asn mutant of hSERT confirm the prediction that Asp437 is a central residue in controlling ion transport.

## Introduction

The human monoamine transporters belong to the family of neurotransmitter sodium symporters. These transporters play a very important role in many processes relating to fundamental brain functions and are therefore of great pharmacological interest [1], [2]. The monoamine transporters are responsible for the reuptake of the neurotransmitters serotonin (5-HT), dopamine, and norepinephrine into presynaptic neurons through the human serotonin transporter (hSERT), the human dopamine transporter (hDAT), and the human norepinephrine transporter, respectively [1].

So far, no high-resolution three-dimensional structure is available of the monoamine transporters. Nevertheless, it has been possible for us and others to create experimentally supported homology models of these transporters [3]–[7], based on the crystal structure of the homologous bacterial leucine transporter (LeuT) [8]. Several high resolution structures of LeuT [8]–[14] have been solved following the first publication, which showed LeuT in an outward-facing partially occluded conformation [8]. However, these do not include an inward-facing conformation of LeuT and it has accordingly not been possible to use LeuT as a template for homology modeling of an inward-facing hSERT. In addition to the LeuT structures, other crystal structures of transporters sharing the “LeuT-fold” have emerged during the last couple of years [15]–[23]. These crystal structures capture the transporters in various states ranging from outward-facing [15], [17], [23] via different partially occluded states [15], [18], [20]–[22] to fully inward-facing conformations [16], [19]. LeuT contains an inherent inverted repeat where TM1–5 is related to TM6–10 by a pseudo C2-axis [8]. This property has been suggested to be an important feature during transport [24]. “LeuT-fold” transporters, including the monoamine transporters, are believed to function by an alternating access mechanism, meaning that the substrate is only exposed to one side of the cell membrane at a time [25]. Modeling-based approaches have explored possible inward-facing states of LeuT taking advantage of this repeat, thereby substantiating the alternating access mechanism of transport [24], [26]. From the two inverted repeats, Forrest *et al.* [24] proposed that the conformational change involved in the transport mechanism is a rocking bundle movement of a four-helix bundle consisting of TM1, TM2, TM6, and TM7 with respect to the scaffold (TM3–5, TM8–10) [24]. A similar mechanism has been described based on crystal structures of Mhp1 where the conformational changes have been described as a hash-motif movement (TM3, TM4, TM8, TM9) with respect to the four-helix bundle (TM1, TM2, TM6, TM7), with TM5 and TM10 functioning as gates during conformational changes [15], [16]. From steered molecular dynamics pulling the leucine substrate out of LeuT, we initially found that the substrate reaches a transient extracellular secondary binding site during transport with lower affinity than found in the central binding pocket [27]. The revelation of a secondary vestibular binding site, was later proposed to function as an actual part of the transport mechanism in LeuT where the second substrate occupying the binding site in the extracellular vestibule functions as a symport effector site [26]. This idea has been further extended to form part of the actual transport mechanism of hDAT containing two substrates based on biased molecular dynamics simulations [28]. The rocking four-helix-bundle model [24], [29] fits well with the observed crystal structure of other proteins containing the “LeuT-fold” [23], [30], however the presence of a secondary actuator site has not been validated through high-resolution structures with multiple occupations of substrates in these transporters. Nevertheless, the binding of inhibitors in the extracellular vestibule [9]–[13] indicates that there might be a secondary binding site here, albeit with low affinity [10], [31].

From substituted cysteine accessibility experiments it was found, that residues Phe88, Ser91, Gly94, Gly273, Ser277, Val281, Thr284, Phe347, Ala441, Glu444, and Thr448 on TM1, TM5, TM6 and TM8 line the cytoplasmic transport pathway in hSERT [24], [32]–[34]. These data on hSERT support the proposed rocking-bundle mechanism for LeuT in which the cytoplasmic pathway is indeed lined by these four helices [24]. Two studies investigating dynamic properties of LeuT have appeared, proposing partially independent movements of the intracellular and extracellular halves of LeuT. Based on fluorescence resonance energy (FRET) [35] and electron paramagnetic resonance (EPR) [36] studies it was suggested that when only ions are bound to LeuT, an outward-facing conformation with a very dynamic extracellular vestibule is established, whilst the addition of substrate restrains dynamics of the extracellular vestibule [36]. Additionally, ions and substrate induce rearrangement of the intracellular gate, with especially TM1 moving [35]. The observed disruption of the extracellular and intracellular gates and the resulting change in conformation of LeuT thus implies that these conserved gates can work partly independent [35], [36].

**Figure 1:**
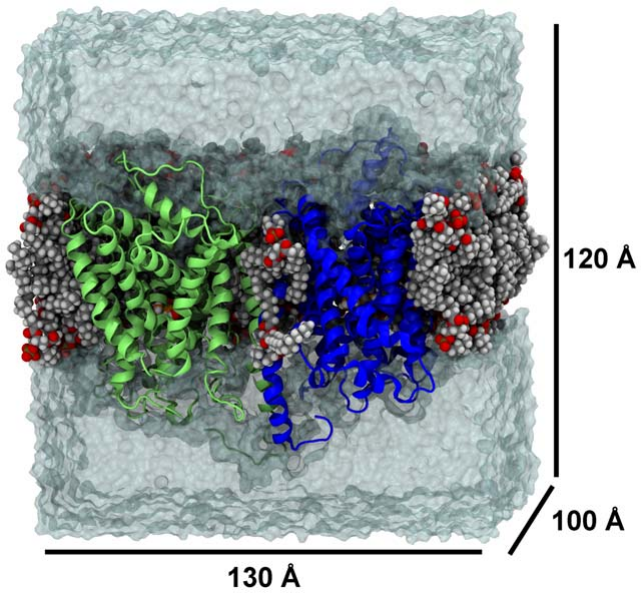
Overview of system. The system simulated is a hSERT dimer embedded in a POPC bilayer solvated with TIP3P [52] water and neutralized with NaCl to a concentration of 0.2 M. The protomers in the dimer are shown in green and blue cartoons, whereas the lipid bilayer is shown in spheres. Water is shown as a transparent surface. The approximate dimensions of the simulation system are included.

We present classical unbiased molecular dynamics simulations of a hSERT homology model [3], [5] in a transport competent state containing ions and the substrate, 5-HT, bound in the central binding pocket in a previously validated orientation [5]. The protein-ligand complexes were studied as dimers reflecting the natural oligomeric state of hSERT, since it has been found from several experiments that hSERT at least must be dimeric for proper functioning in cells [37]–[39], see Figure 1. From simulations of the 5-HT/ions protein dimer, specific movements leading to an inward-facing conformation of hSERT were detected, followed by transport of the sodium ion bound to the Na2-site.

To test the proposed two-substrate mechanism [26], [28], we simulated dimeric hSERT containing both a substrate in the central binding pocket (S1-site) and the extracellular secondary site (S2-site) of both monomers. These molecular dynamics simulations did not result in any transport events; rather they showed indications of dissociation of the weakly bound secondary substrate towards the extracellular side. Finally, the computational predictions were confirmed by experiments on a single-point mutant, Asp437Asn, of hSERT altering the Na2 site. These revealed that release of the Na^+^-ion from the Na2-site may be associated with the initiation of the conformational changes of the transporter towards an inward-facing conformation.

## Results

It was recently shown that more efficient sampling may be achieved through several repeats of a molecular dynamics simulation than from one very long simulation [40]. Therefore, we decided to prepare five repeats of all systems studied, entailing a total of 10 protein-ligand trajectories for analysis due to the dimeric nature of hSERT [37]–[39]. Simulations of 5-HT bound to hSERT, **Sim1–10**, are referred to as 5-HT/ions simulations throughout the paper. **Sim1–5** corresponds to monomer A in the five repeats while **Sim6–10** reflects the dynamics of monomer B of the five repeated simulations of dimeric hSERT. For a complete overview of all simulations, please see Supplementary Information (SI), Table S1. From the five repeats of the 5-HT/ions protein dimer, each of 100 ns simulation, we were able to identify specific movements leading to an inward-facing conformation of hSERT. Prolonged simulations of 5-HT bound hSERT, five repeats each of 50 ns (**Sim8a–e**), started from the identified mostly inward-facing conformation sampled in **Sim8**, showed complete ion transport and early events of substrate release. To test the proposed two-substrate mechanism [26], [28], similar simulations, five repeats of each 50 ns, were carried out of dimeric hSERT (**Sim11–20**) containing a substrate in the central binding pocket (S1-site) and a second substrate in the vestibular S2-site. Here **Sim11–15** corresponds to the trajectories sampled for monomer A and **Sim16–20** to monomer B in the five repeats. No indication of transport was observed from these molecular dynamics simulations, but indications of dissociation of the secondary substrate towards the extracellular side were detected. Finally, simulations of an apo homology model of hSERT with ions bound were carried out for reference (**Sim21–30**, referred to as apo/ions simulations). **Sim21–25** reflects the dynamics of monomer A, while **Sim26–30** describes dynamics of monomer B in the five repeats. Among others, **Sim26** of the apo/ions simulations showed indication of an increased opening towards the extracellular side, and is accordingly used for comparison throughout the paper.

### Stability of the System during Molecular Dynamics Simulations

The overall stability of the protein systems were monitored through root-mean-square deviation (RMSD) and fluctuations (RMSF). The systems remain stable during the 100 ns simulations both in the 5-HT/ions, **Sim1–10**, and in the apo/ions simulations, **Sim21–30** (Figure S1), and equilibrated structures are reached after few nanoseconds of simulation time based on the plateauing of the RMSD-curves.

**Figure 2:**
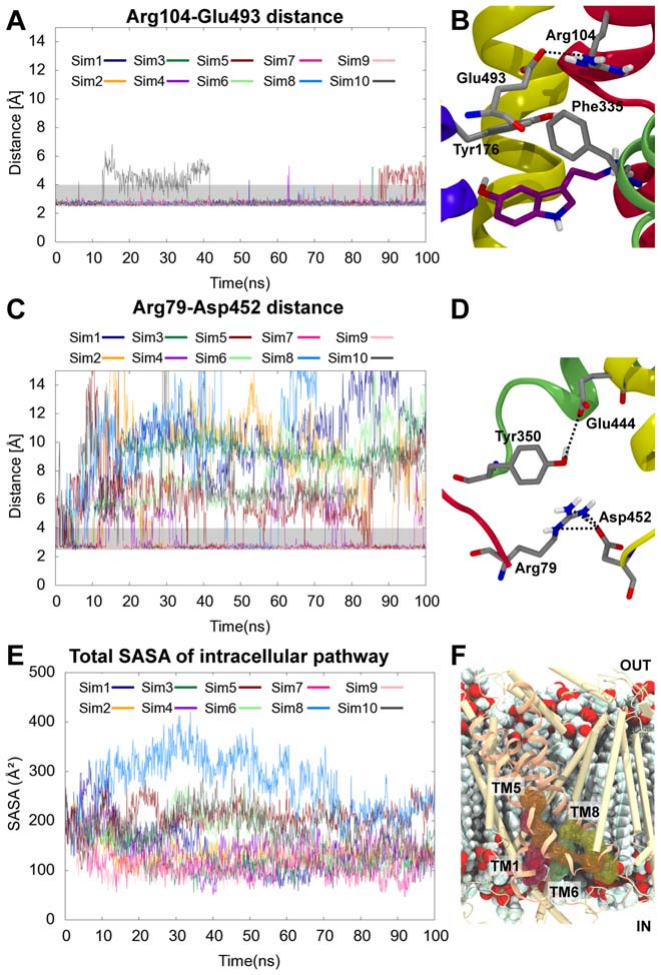
Gate interactions and intracellular pathway. Transition to the inward-facing conformation causes disruption of the intracellular gate but not the extracellular gate, and solvation of the cytoplasmic pathway to the substrate site occurs. A. The extracellular gate interactions remain stable throughout simulation as illustrated by the depicted shortest distance between the carboxylate oxygen atoms of Glu493 and guanidinium nitrogen atoms of Arg104. B. Arg104 and Glu493 form an ionic interaction and contribute to the extracellular gate. Tyr176 and Phe335 form an aromatic lid on top of the central binding pocket lined by TM1 (red), TM3 (blue), TM6 (green), and TM8 (yellow). Protein side chains are shown in gray, and the 5-HT substrate is colored by atom type with carbons in purple. C. The shortest distance between Asp452 carboxylate oxygen atoms and Arg79 guadinium nitrogen atoms in the intracellular gate are seen. D. In the intracellular gate, Arg79 and Asp452 form an ionic interaction, and Tyr350 and Glu444 interacts via a hydrogen bond. The N-terminal is shown in red, TM6 in green and TM8 in yellow. Protein side chains are shown in gray sticks. E. Calculated SASAs of the proposed cytoplasmic pathway residues (Phe88, Ser91, Gly94, Gly273, Ser277, Val281, Thr284, Phe347, Ala441, Glu444, and Thr448) are shown. The SASA of **Sim8** (light blue line) increases from around 200 Å^2^ to 400 Å^2^ after 35 ns of simulation. F. Position of cytoplasmic pathway residues in hSERT. TM1, TM5, TM6 and TM8 are shown in light salmon. The remaining TMs are shown in beige. The residues found experimentally to line the cytoplasmic pathway [24], [32]–[34] are displayed in sticks surrounded by transparent surfaces and colored with those from TM1 in red, TM5 in orange, TM6 in green and TM8 in yellow. The POPC bilayer is shown in spheres and colored by atom type with light blue carbons.

The local dynamics of the 5-HT/ions simulations were further explored by distance measurements of the extracellular and intracellular gates in hSERT. The extracellular gates are composed of the salt-bridge between Arg104 and Glu493 and the aromatic lid composed of Tyr176 and Phe335, shielding the central 5-HT binding site from the extracellular vestibule [8], see Figure 2A and B. Additionally, the intracellular gate interactions composed of an ionic interaction between Arg79 and Glu452 and a hydrogen bonding interaction between Tyr350 and Glu444 were followed to explore the dynamics of the cytoplasmic part of the transporter [41], see Figure 2C and D. The salt-bridge of the extracellular gate (Arg104-Glu493) was already found in the 5-HT/ions starting structure and it remains stable in all the systems. Only minor fluctuations are observed in **Sim10** where the interaction is lost after approximately 12 ns, but reforms after 40 ns. In the apo/ions system (**Sim21–30**) the extracellular gate fluctuates much more than in the 5-HT/ions system (Figure S3). A similar behavior was recently detected for LeuT with ions bound, where it was found that an apo/ions LeuT is more flexible at the extracellular gates than substrate-bound LeuT [36].

The solvent accessible surface area (SASA) of the aromatic lid (Tyr176 and Phe335) decreases during the simulation compared to the starting structure in the 5-HT/ions simulations (Figure S2). This may indicate a tightening of the extracellular vestibule similar to what was inferred from EPR measurements upon substrate binding to LeuT [35]. The SASA of the aromatic lid in the apo/ions systems increases much more than observed in the 5-HT/ions system (Figure S2 and S3). This further indicates that the extracellular cavity in the apo/ions system is more flexible and open than observed for the substrate bound transporter.

The intracellular gate in the 5-HT/ions simulations is found to be very unstable in virtually all of the simulations (**Sim1–10**) with disruption of the Arg79-Asp452 ionic interaction occurring almost immediately in **Sim1–10**, see Figure 2C and D. Also, the distance of the interaction partners, Tyr350 and Glu444, was monitored as an indication of cytoplasmic gate stability. This interaction remains stable in less than half of the 5-HT/ions simulations (Figure S2) suggesting that the substrate bound hSERT is transitioning towards an inward-facing conformation simultaneously with a closure of the extracellular cavity. In the apo/ions system, the intracellular gate interactions also break in most of the cases (Figure S3). This is comparable to the observation from FRET studies of LeuT, showing that the presence of ions and substrate cause rearrangement in the intracellular gate [35].

In order to monitor whether an intracellular opening of the transporter occurs concurrently with the disruption of the intracellular gate during simulation, SASA calculations for residues previously proposed to line the cytoplasmic pathway of hSERT [24], [32]–[34] were performed. The total SASA of these residues; Phe88, Ser91, Gly94, Gly273, Ser277, Val281, Thr284, Phe347, Ala441, Glu444, and Thr448, is illustrated in Figure 2E, and Figure 2F shows how these residues indeed line the cytoplasmic vestibule of hSERT identified in our simulations. It is evident that the solvent accessibility increases in some of the simulations, especially in **Sim8** where a dramatic increase is observed around 35 ns, indicating substantial solvent exposure of the cytoplasmic pathway. As can be seen from Figure 2E, an increase in SASA is a general trend in many of the simulations, however most pronounced in **Sim8**, and this system is therefore the main focus throughout the remaining part of this paper.

The number of water molecules interacting with the intracellular pathway residues in **Sim8** was counted during the trajectory. It is found that the number of water molecules within 3 and 4 Å of the cytoplasmic pathway residues mentioned above increases rapidly from around 40 interactions to more than 70 between approximately 25 and 40 ns (Figure S4). This further supports the findings from the SASA calculations, signifying an increased solvent accessibility of the cytoplasmic pathway residues.

### Solvation of the Central Binding Pocket, and Extracellular and Intracellular Vestibules

**Figure 3:**
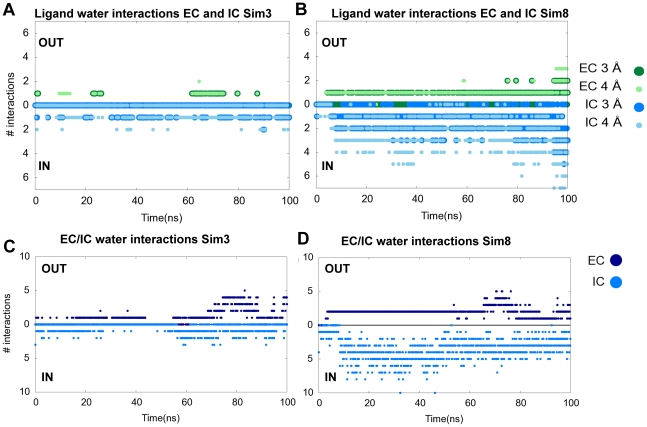
Comparison of solvation from the extracellular and intracellular sides. In **Sim8** the transition to the inward-facing conformation causes the intracellular pathway and substrate to be solvated by water from the cytoplasm. Solvation patterns of the ligand are shown as the number of water molecules within 3 Å (dark green) and 4 Å (light green) from the extracellular (top) and intracellular cavities (bottom) in system **Sim3** (A) and system **Sim8** (B). Number of water molecules located in the extracellular (EC, dark blue, top) and the intracellular side (IC, light blue, bottom) in system **Sim3** (C) and system **Sim8** (D). Data are extracted from 1000 snapshots during the trajectories.

The degree of solvation of the ligand in the binding pocket was investigated to substantiate whether the simulated inward-facing conformation of the transporter reaches a state that would be consistent with substrate translocation. For comparison to **Sim8**, a trajectory not exploring large conformational changes during the simulation was chosen. Since **Sim3** does not undergo large conformational changes and is the other monomer of the dimer system containing **Sim8**, this trajectory was chosen for analysis. However the trends from **Sim3** are similar to other trajectories with no or little conformational changes. The number of interacting water molecules within 3 and 4 Å of the substrate is depicted in Figure 3A and B, for **Sim3** and **Sim8**, respectively, as the ones originating from the extracellular cavity (EC) (z>0 Å) and the ones from the intracellular cavity (IC) (z<0 Å), where z equals 0 Å is at the center of mass of the substrate. It is seen that the number of water molecules interacting with the ligand from the intracellular site in **Sim8** is larger than the number of water molecules interacting with it from the extracellular side (Figure 3B), whereas the number of water molecules interacting with the ligand in **Sim3** from both sides remains low (Figure 3A) since this transporter does not undergo large conformational changes. This indicates that a considerable ligand solvation appears in **Sim8**, and that these water interactions mostly stem from the intracellular pathway, whereas the binding site in **Sim3** persists in a more or less occluded state throughout the 100 ns simulation.

The numbers of water molecules occupying the extracellular and intracellular cavities, respectively were calculated throughout the **Sim3** and **Sim8** simulations similarly to Shaikh and Tajkhorshid [42], and are depicted in Figure 3C and D. The number of water molecules in the extracellular cavity is defined as water molecules lying between 2<z<15 Å, and concurrently within 10 Å of residue Phe335 and TM1, TM6, TM8 and TM10. The intracellular cavity is defined as water molecules within 10 Å of Phe341 and TM1, TM6, TM8 and TM5. The number of water molecules in the intracellular cavity of **Sim8** is larger compared to the extracellular cavity, but also compared to both extracellular and intracellular cavities in **Sim3**. This supports that **Sim8** reaches a truly inward-facing conformation with the extracellular cavity closed and a maximum of intracellular cavity solvation obtained after approximately 25–40 ns. In addition, we observe that the number of water molecules in the intracellular cavity decreases slightly after approximately 50 ns of simulation. This indicates that the transporter passes through different conformational stages, and still remains dynamic. The observed increase in solvation between approximately 25 and 40 ns in **Sim8** is accordingly not an indication of an unstable transporter. In the apo/ions system, **Sim26**, where an opening to the extracellular site is observed, the number of water molecules in the extracellular cavity is generally much greater than within the intracellular cavity, see Figure S3, and also compared to the 5-HT/ions systems.

### Na2 Solvation and Transport

**Figure 4:**
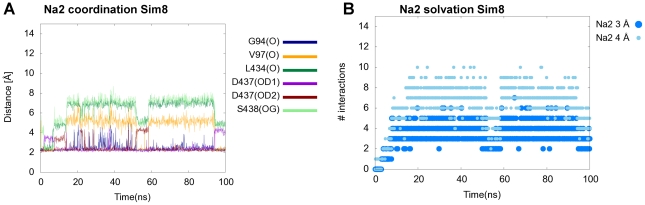
Na2 coordination and solvation. Na2-ion coordination is less stringent upon transition to the inward-facing conformation. A. Traces represent distances in **Sim8** between the Na2-ion and the initial coordination residues in the Na2-site. The number of residues coordinating the Na2-ion decreases during the simulation with Asp437 and Gly94 being the only continuous coordination partners. B. The Na2-ion becomes solvated upon transition to the inward-facing conformation. Dots represent the number of water molecules interacting with the Na2-ion in **Sim8**. After a few ns the number of water molecules within 3 Å increase to 4–5, which then substitute for the coordination from the protein.

**Figure 5:**
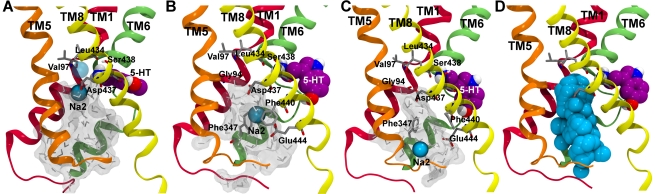
Na2 transport in hSERT. The transition to the inward-facing conformation opens up a pathway towards the cytoplasm for the Na2-ion lined by TM1 (red), TM5 (orange), TM6 (green), and TM8 (yellow). The 5-HT substrate is shown in spheres and colored by atom type, with carbons in purple. Protein residues are shown in gray sticks, and the Na2-ion is shown as a cyan sphere with the initial position of the Na2-ion indicated as a transparent light blue sphere. Water molecules found in the intracellular cavity is indicated by light gray sticks and a transparent surface. A. The snapshot after 33.9 ns molecular dynamics simulation in **Sim8** where the Na2-ion is located just below the initial binding pocket formed by Ser438, Leu434, Val97, Gly94, and Asp437. The only coordination partners remaining are Asp437 and Gly94, which are located in the bottom of the ion binding site. B. Molecular dynamics snapshot after 75.4 ns (33.9 ns snapshot from **Sim8** and additional 41.5 ns in **Sim8b**). The Na2-ion is partly transported and located between the two aromatic residues Phe347 and Phe440 and coordinated by the acidic residue Glu444. C. The Na2-ion is completely transported after 80.4 ns (33.9 ns in **Sim8** and additional 46.5 ns in **Sim8b**) and is fully solvated by water and does not possess any interactions with the protein. D. The monitored positions of the Na2-ion in **Sim8b** are displayed as cyan spheres during the additional 50 ns of simulation started from the **Sim8** snapshot. The spheres indicate the transport pathway of the Na2-ion from the central ion binding site to the cytoplasm. It can be observed that TM1, TM5, TM6, and TM8 are lining the transport pathway.

When evaluating the ion coordination during the simulation it is observed that the sodium ion occupying the Na1-site as well as the chloride ion both remain stably coordinated throughout the 100 ns of simulation in **Sim8** (Figure S5). This is in sharp contrast to the observed changes in the coordination of the sodium ion occupying the Na2-site in **Sim8** where only Asp437 and Gly94 coordinations are maintained, see Figure 4A. After a few nanoseconds of simulation, the number of water molecules within 3 Å of the Na2-ion increases to 4–5 water molecules in **Sim8**, see Figure 4B. This increase in the number of water interactions in addition to the decrease in protein coordination number occurs because the sodium ion leaves the Na2-site, with a side chain rotation of Asp437 being the largest protein movements involved in the translocation (Figure S6). Hereby, Asp437 is able to retain the coordination with the Na2-ion as the final Na2-site gatekeeper. This is similar to a recently observed rotation of the side chain of Thr354, the corresponding residue in LeuT, from simulations by Zhao and Noskov [43]. The Na2-ion is accordingly positioned just below the initial ion binding site as seen in Figure 5A. After approximately 50 ns of simulation a drop in the protein coordination distances and the number of water interactions with the Na2-ion is observed in **Sim8** (Figure 4A and B). This shift is caused by a rapid Asp437 side chain rotation resulting in the Na2-ion moving back into the original ion binding site re-establishing most of the native ion coordinations. The Na2-ion remains in the original Na2-site for approximately 10 ns, after which it jumps back into the pocket below the binding site. From the coordination pattern and water interactions it is evident that water molecules substitutes for some of the native protein coordination partners. The Na2-ion site is accordingly being almost fully solvated, with a water wire connecting the site directly to the cytoplasm, see Figure 5A.

To explore possible transport events, additional simulations were performed using the most-open inward-facing conformation identified after 33.9 ns molecular dynamics simulation in **Sim8**. The inward-facing structure was chosen based on the snapshot receiving the highest calculated SASA of the intracellular pathway. This snapshot was used as a starting structure for five new simulations after a minimization. The prolonged simulations were each run for 50 ns (**Sim8a–e**). In three out of these five simulations, the Na2-ion becomes fully released via the cytoplasmic pathway. One example of Na2-ion release in **Sim8b** is illustrated in Figure 5. The first step corresponds to the Na2-ion being located just below the original Na2-site as described for **Sim8** with only Asp437 and Gly94 remaining as coordination partners, Figure 5A. After staying almost stable in this site for approximately 41 ns in **Sim8b**, the Na2-ion loses the interactions with the native coordination partners and receives electrostatic interactions with Glu444 and cation-π interactions with two aromatic residues, namely Phe440 and Phe347, see Figure 5B. The Na2-ion is eventually further translocated to be fully solvated after 46 ns only experiencing water interactions, Figure 5C. The Na^+^ release follows the proposed [24], [32]–[34] cytoplasmic pathway between TM1, TM5, TM6, and TM8 during transport which can be visualized from the overlay of the positions sampled of the Na2-ion during the simulation (Figure 5D). Additionally, the substrate also shows early events of transport. The substrate slides deeper into the binding site, and establishes an interaction between the 5-hydroxyl group and Ala441 located deep in the central binding pocket instead of the Ser438 backbone (Figure 5). The substrate rotates and slides downwards between TM6 and TM8 interacting with Ala441, which also forms part of the cytoplasmic pathway [24], [32]–[34]. The substrate therefore is in close proximity to the water molecules that are solvating and accordingly facilitating the transport of the Na2-ion, and is on its way to becoming fully solvated and transported via the cytoplasmic pathway.

### Biochemical Exploration of Na2-ion Binding Affinity and Release

**Figure 6:**
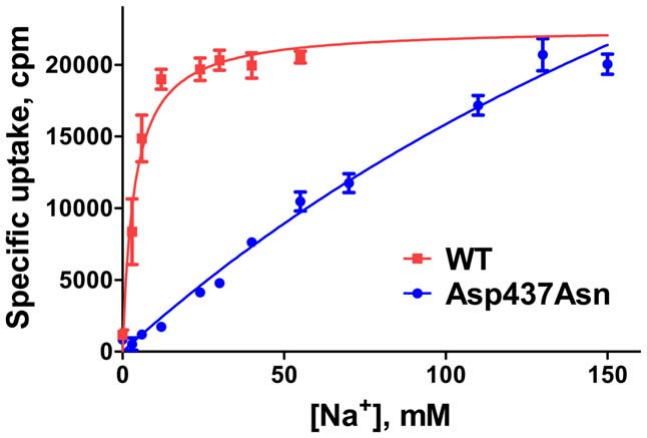
Effects of Asp437Asn mutation. Mutation of Asp437 to Asn in the Na2-site severely compromises the apparent transport affinity for sodium. Radiotracer uptake of 10 µM 5-HT into HEK293MSR cells transiently transfected with hSERT wt or the Asp437Asn mutant reveals that removal of the sodium coordinating side chain of residue 437 does not affect maximum transport rates negatively whereas sodium affinity is significantly impaired. NaCl is substituted with N-methyl-D-glucamine. Data is fitted to Michaelis-Menten kinetics.

From the molecular dynamics simulations, Asp437 was identified as an important interaction partner of the Na2-ion and was predicted to be the final interaction partner when the sodium ion is released from the Na2-site. Therefore, Asp437 may play a role in both initial binding of Na^+^ and govern the rate of release of the Na2-ion and consequently the subsequent release of substrate. To verify the crucial role of Asp437 in coordination of the Na2-ion and to determine the possible role of the Na2-ion in driving the translocation implied by the molecular dynamics simulations, a radiotracer uptake experiment in mammalian cells transiently transfected with wild type (wt) hSERT and an Asp437Asn single-point mutant of hSERT at varying concentrations of Na^+^ was performed, enabling determination of the K_M_ for Na^+^ during transport, see Figure 6.

The Asp437Asn mutation is sterically conservative but removes the charged acidic side chain coordinating the sodium ion. This interaction was implied from the simulations to be the most important one in coordination of the Na2-ion. Apparent 5-HT transport affinity is decreased 12-fold (p = 0.0487, paired t-test) in the Asp437Asn mutant compared to wt hSERT, indicating that the incomplete saturation of the Na2-ion in the Asp437Asn mutant even at 150 mM sodium concentration severely impacts 5-HT binding.

Mutating Asp437 to Asn has a considerable influence on the functional sodium affinity whereas apparent substrate affinity and V_max_ does not seem to be affected significantly, see data in Table 1. Transport rates in the Asp437Asn mutant are comparable to wt hSERT at 150 mM sodium ion concentration (p = 0.301, two-tailed paired t-test) but do not seem to saturate in isotonic buffer for the Asp437Asn mutant. By extrapolation, the Asp437Asn mutant would have maximum uptake rates exceeding wt hSERT at hypertonic sodium concentrations. The affinity for sodium in the Asp437Asn mutant is so low that sodium binding does not saturate at isotonic conditions making it difficult to determine the true K_M_ and V_max_ for Na^+^ in cell-based uptake experiments reliably, see Figure 6. However, by non-linear regression analysis, the K_M_ is calculated to be in excess of 180 mM for the Asp437Asn mutant. If saturation is assumed to take place immediately above the highest sodium concentration tested, then the most conservative estimate results in a K_M_ for Na^+^ at or above 73 mM (SEM ±2.6 mM), thus the mutation decreases the apparent transport affinity of sodium significantly (p<0.0001, unpaired t-test) by at least 13-fold compared to wt hSERT (K_M_ = 5.9 mM, SEM ±0.9 mM) but likely by much more.

**Table 1:** Kinetic parameters from radiotracer uptake experiments.

	hSERT wt	Asp437Asn
K_M, Na+_, mM	5.893 [3.751 ; 8.036 ]	≥72.67^a^ [65.40 ; 79.93]
V_max,Na+_	100%	143%^b^ [42.39% ; 243.7%]
K_M,5HT_, µM	0.2974[0.02283 ; 0.5720]	3.315^c^[0.4889 ; 6.140]

a *P<0.0001, n = 5, Asp437Asn did not saturate within the sodium concentration used.*

b *P = 0.3008, n = 5.*

c *P = 0.0102, n = 3.*

Kinetic parameters from radiotracer uptake into HEK293MSR cells transiently transfected with hSERT wt or Asp437Asn. Bracketed values represent 95% confidence limits. Kinetic parameters for hSERT wt and Asp437Asn were compared for statistical significance using students t-test.

### Pore Formation in the Inward-Facing hSERT Conformation

**Figure 7:**
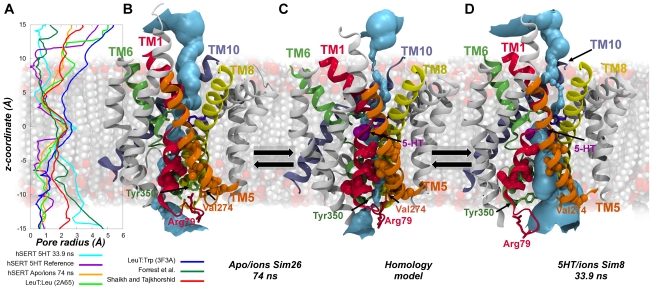
Comparison of pores in outward, occluded and inward-facing hSERT. The pore running through the transporter is found to open dramatically towards the cytoplasm during the simulations reflecting an inward-facing transporter. The pore diameter is measured utilizing the HOLE plug-in for VMD [44], [45]. A. Comparison of the pore diameter through the protein is shown for the inward-facing 5-HT/ions molecular dynamics snapshot at 33.9 ns in **Sim8** (cyan), the hSERT homology model (purple), an outward-facing apo/ions hSERT molecular dynamics snapshot from **Sim26** (yellow), open-occluded LeuT (PDB:2A65, light green) [8], tryptophan bound outward-facing LeuT (PDB:3F3A, dark blue) [9], inward-facing LeuT by Forrest *et al.* (dark green) [24] and inward-facing LeuT by Shaikh and Tajkhorshid (red) [42]. B–D. Molecular illustrations of the pore running through hSERT as found in **Sim26** of the apo/ions simulation after 74 ns (B), in the hSERT 5-HT/ions homology model (C), and in the hSERT 5-HT/ions molecular dynamics snapshot at 33.9 ns in **Sim8** (D). The pore is shown in light blue surface in B–D. TM1, TM5, TM6, TM8, and TM10 are colored in red, orange, green, yellow, and ice blue respectively, while TM2, TM3, TM4, TM7, and TM9 are shown in gray. The residues that have been shown to line the cytoplasmic pathway of hSERT from substituted cysteine accessibility scanning [24], [32]–[34] are shown in spheres using the colors corresponding to the TM they belong to. All residues experimentally shown to increase accessibility upon inward-facing conformation are included. The residues involved in the intracellular gate (Arg79, Val274, Tyr350, Asp452 and Glu444) are shown in sticks using the same color as the TMs they belong to.

The program HOLE [44], [45] was applied to estimate the radius of the channel formed in the transporter in the inward-facing conformation, and is shown in Figure 7. Here, the snapshot collected from **Sim8** after 33.9 ns is compared to the validated homology model of hSERT containing 5-HT [3], [5], an outward-facing snapshot of hSERT from the apo/ions simulation (**Sim26**), in addition to LeuT crystal structures in the outward-occluded state [8] and the outward-open conformation [9], as well as to the two proposed models of inward-facing LeuT by Forrest *et al.* [24] and Shaikh and Tajkhorshid [42]. From the radius calculations in Figure 7A, it is clear that the snapshot from **Sim8** after 33.9 ns has a much wider intracellular pore compared to both the homology model of hSERT, the outward-facing hSERT conformation extracted from the apo/ions simulation, **Sim26**, and the two LeuT crystal structures [8], [9]. Upon comparison with the two proposed inward-facing LeuT models build based on the inverted repeats [24] and from homology modeling followed by targeted molecular dynamics simulations [42], it is seen that the pore observed in **Sim8** after 33.9 ns is comparable to the LeuT pores found in the two studies close to the central binding site, but wider closer to the cytoplasm (Figure 7A). Furthermore, the extracellular part of the pore is more closed in this snapshot from **Sim8** than observed in both the two LeuT crystal structures [8], [9] and in the outward-facing apo/ions simulation (**Sim26**), as well as in the inward-facing model of LeuT proposed by Shaikh and Tajkhorshid [42]. This latter model seems to represent a state where pores have similar radii on the extracellular and intracellular side, which could possibly be induced by the biasing method applied when generating the model [42]. The 5-HT/ions simulation snapshot after 33.9 ns in **Sim8** shows an extracellular pore topology that is comparable to the hSERT homology model [3], [5] as well as to the inward-facing LeuT model reported by Forrest *et al.* [24]. From the molecular illustration in Figure 7 of the pores in the outward-facing apo/ions hSERT snapshot in **Sim26** (Figure 7B), the homology model of hSERT (Figure 7C), and the snapshot of **Sim8** after 33.9 ns (Figure 7D), it is evident that the pore diameter of the intracellular cavity increases dramatically in the latter. The snapshot of the apo/ions simulations in **Sim26** is almost completely closed towards the cytoplasm, whereas this structure shows a greater radius towards the extracellular side compared to the two other hSERT structures. Also the homology model of hSERT is almost closed completely to the intracellular side when compared to the 33.9 ns snapshot of **Sim8**. The residues shown to line the cytoplasmic pathway [24], [32]–[34] are illustrated as spheres and it is apparent from Figure 7D that these residues do indeed line the pore being formed in **Sim8**. From comparison of the three conformations it is obvious, that especially TM1a (red ribbons) and TM6b (green ribbons) induce the opening of the cytoplasmic pathway. This can also be shown from the breakage of the interactions within the intracellular gate network with especially Arg79 and Tyr350 from TM1 and TM6 respectively moving considerably causing this intracellular gate disruption. This observation agrees well with the proposed transport mechanism involving especially movements of the four-helix bundle with respect to the remaining protein [24], [29].

### Conformational Changes during Transport

**Figure 8:**
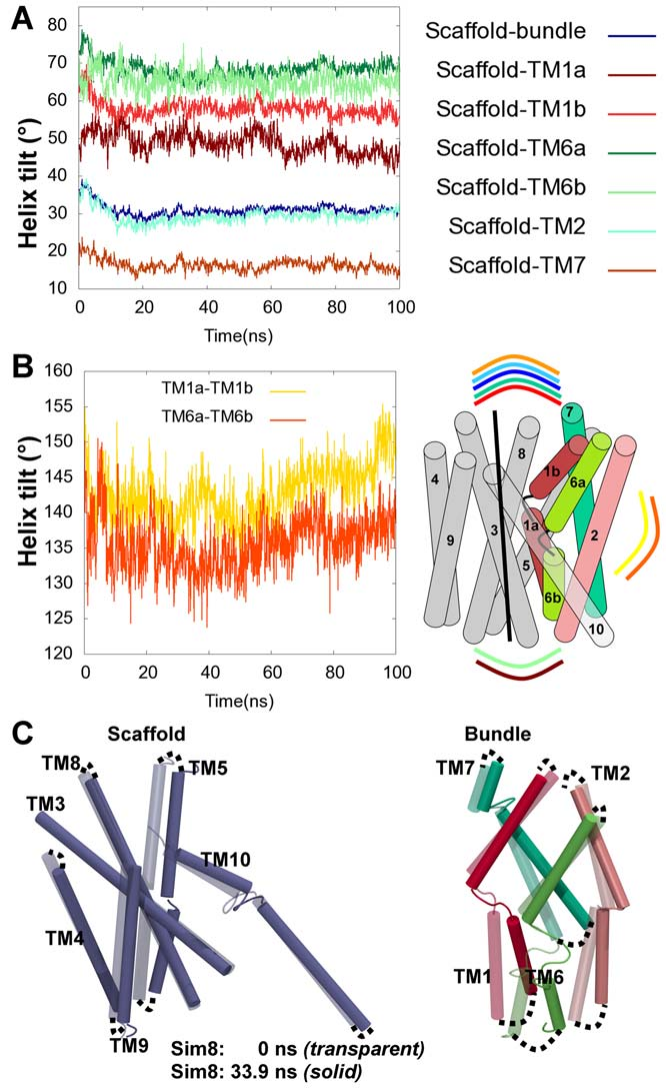
Helix movements leading to conformational changes. The intracellular parts of TM1 and TM6 are the most flexible helices in the transition to the inward-facing conformation. TM helix movements in the **Sim8** system are represented by the relative movement compared to the scaffold and the starting structure. The simulations disclose a partly un-coupled movement of the two halves of the unwound helices, TM1 and TM6. A. The angles between scaffold/bundle (dark blue), scaffold/TM1a (dark red), scaffold/TM1b (red), scaffold/TM6b (dark green), scaffold/TM6b (light-green), scaffold/TM2 (cyan), scaffold/TM7 (dark-orange) are depicted along the trajectory in **Sim8**. B) The angles between TM1a/TM1b (yellow), TM6a/TM6b (orange) and the protein features between which the helix-tilts are measured are shown schematically in colors corresponding to the lines in the graphs. C. The movements of the different protein parts between the starting structure of **Sim8** and the snapshot after 33.9 ns within the scaffold (left) and the bundle (right) are depicted. The intracellular part of the four-helix bundle (red and green helices) is moving the most. The movement is illustrated based on a structural alignment on TM3 and TM8 forming part of the hash-motif [42].

In order to investigate which movements are involved in the conformational changes from the initial outward-facing occluded state of hSERT to the inward-facing conformation, the tilt angles between the scaffold (TM3–5 and TM8–10) and the four-helix bundle (TM1, TM2, TM6, and TM7) were calculated (Figure 8). It can be observed, that the angle between the scaffold and the bundle (denoted as scaffold/bundle angle in Figure 8A shown in dark blue) and the individual angles between the scaffold and the bundle (TM1b, TM2, TM6a and TM7) behave very similarly during the trajectory of **Sim8** (Figure 8A). These angles all decrease during the first ∼20 ns, followed by a long plateau phase, indicating that an equilibrium state has been reached. The measured decrease in tilt angle observed in these parts indicates an overall movement of the extracellular part of the four-helix bundle part towards the scaffold. This further supports a closing of the extracellular gate and an opening of the intracellular cavity. A slightly different behavior is observed for the angles between the scaffold and the cytoplasmic halves of the two centrally unwound helices, scaffold/TM1a (burgundy red) and scaffold/TM6b (light green). These two angles are found to initially increase and generally they fluctuate more than the other angles measured, further underlining that these two helixes are important players in the observed conformational change of hSERT. When the angle between the two parts of TM1 (yellow) and TM6 (light orange) around their unwound parts are measured, it becomes evident that the two halves of these helices are indeed internally flexible (Figure 8B). They do not necessarily move as a rigid domain as implied in the helix-bundle mechanism [24], [29], but may possess an internal hinge. This observation is in agreement with the recent studies of the dynamic behavior of LeuT based on FRET [35] studies where TM1 undergo rearrangements during conformational transitions. The overall movements that occur between the initial structures (transparent) and the snapshot of the inward-facing conformation (solid color) in **Sim8** are revealed in Figure 8C. The largest movements occur between the four-helix bundle (red and green cylinders) and the scaffold, where the individual scaffold TMs remain stable during the simulations (Figure 8C, light blue cylinders). Within the bundle, the intracellular part moves considerably more than the extracellular part of these helices. The unwound TM1 and TM6 could accordingly be involved in conformational changes during transport by a hinge type of motion where the transporter is closed to the extracellular side by large global movements, whereas the intracellular opening is accomplished by hinge type motions of TM1a and TM6b. The transporter could thereby be functioning with partly un-concerted movements between the two parts of the bundle according to the rocking-bundle mechanism and in agreement with recent studies on LeuT [35], [36].

**Figure 9:**
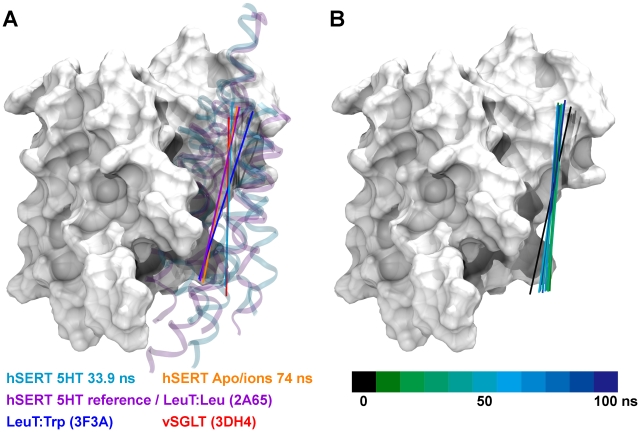
Conformational states and sampling of the four-helix bundle. The hSERT structures adapt conformational stages comparable with experimentally known outward-occluded and inward-facing states. During the trajectory in **Sim8** various conformational stages are sampled, with the largest movement occurring on the intracellular side of the four-helix bundle. A. The scaffold is illustrated by light gray surface with the last ten residues of TM10 omitted for clarity. The vectors representing the axis of the four-helix bundle are shown by colored lines. The four-helix bundle of the inward-facing conformation of hSERT obtained from the 33.9 ns snapshot in **Sim8** and the reference homology model are shown in ribbons colored in cyan and purple, respectively. The location of the four-helix bundle of the reference homology model (purple) and the outward-facing conformation of hSERT from the apo/ions **Sim26** 74 ns snapshot (orange) are very similar to what was observed from the crystal structure of LeuT with leucine bound (not shown as it overlays perfectly with the reference homology model in purple). The outward-facing conformation of LeuT with tryptophan bound (dark blue) has the vector of the four-helix bundle further displaced from the extracellular surface of the scaffold than any of the other structures. The inward-facing conformation of hSERT from **Sim8** (cyan) is as open to the intracellular side as the inward-facing conformation of vSGLT (red) in the crystal structure. B. The scaffold is illustrated similar to in (A) and for clarity the bundle is removed. The vectors corresponding to arrangements of the four-helix bundle during the entire trajectory of **Sim8** are illustrated using snapshots collected every 10 ns starting with the black vector changing from green to blue. The four-helix bundle sample different conformations during the simulation. The arrangement of the four-helix bundle starts from a conformation similar to the outward-occluded LeuT structure and reaches an arrangement similar to the conformation seen in inward-facing vSGLT. The largest movements of the four-helix bundle are seen to occur at the intracellular side.

The sampling of the different conformational states in **Sim8** was compared to known conformational states of similar proteins, using an analysis of the angles between the scaffold and the bundle, similar to what was recently done in a comparative study of LeuT-fold proteins [29]. The outward-facing conformation is exemplified by the outward facing LeuT crystal structure containing the competitive inhibitor tryptophan [9]. The outward-occluded state is illustrated by the leucine bound LeuT crystal structure and the hSERT starting structure, while the inward-facing state is measured relatively to the crystal structure of the *Vibrio parahaemolyticus* sodium galactose transporter (vSGLT) [21]. The vector defined by the four-helix bundle in the simulated hSERT structures in relation to the different conformational stages observed from crystal structures of LeuT and vSGLT is illustrated in Figure 9A. In both the reference homology model of hSERT and the apo/ions snapshot of **Sim26** the four-helix bundle vectors overlay with that of the crystal structure of LeuT in the outward occluded state. None of the hSERT structures reaches a conformation as fully outward-facing as observed in the LeuT∶Trp crystal structure. The inward-facing snapshot of hSERT from **Sim8** is observed to adapt a fully inward-open conformation since the relative orientation of the four-helix bundle vector in this snapshot overlay with that from vSGLT crystal structure. In Figure 9B the sampling of conformations along the trajectory in **Sim8** is shown. Again the starting structure is similar to the outward-occluded LeuT structure from which it was built. The transporter changes from the outward-occluded conformation to an inward-facing conformation followed by some closure of the four-helix bundle back towards the scaffold. Similar to what was observed from Figure 8C, it can be seen that the largest movements of the four-helix bundle occur at the intracellular side, while the extracellular side packs towards the scaffold where it remains stable during the 100 ns of simulation.

### Is a Second Substrate Necessary for Transport in hSERT?

Experimental studies have shown that LeuT only contains a single high affinity binding site [31]. However, studies based on biased molecular dynamics simulations have suggested that a second substrate in the extracellular vestibular S2-site is necessary for transport to occur [26], [28]. In these studies, a biasing pulling force was incorporated ensuring movement of the substrate towards the intracellular side. Additionally the sodium ion occupying the Na2-site was deleted prior to the simulation of leucine transport in LeuT. We docked 5-HT into the proposed secondary actuator site in the extracellular cavity [26] while keeping another 5-HT molecule in the central binding pocket and retaining the three ions to explore the effect on the dynamics of hSERT. To explore possible early signs of transport events in such a system of hSERT, the best pose from this docking was subjected to five repeats of 50 ns unbiased molecular dynamics simulations resulting in ten trajectories, **Sim11–20**, due to the oligomeric nature of the transporter [46]. Neither transport of the substrate in the primary binding pocket nor of any of the ions was observed during these simulations, which is in contrast to what was suggested for leucine in LeuT and dopamine in hDAT [26], [28]. In contrast the 5-HT located in the secondary site is observed to move away from the S2-site towards the extracellular side in the simulations. The amount of water entering by the cytoplasmic pathway to the central binding site is also much smaller in all of these simulations compared to what was observed in the identified inward-facing conformation of hSERT in **Sim8**. This strongly suggests that hSERT does not need two substrates to facilitate transport.

## Discussion

In this study we have discovered the formation of an inward-facing conformation of hSERT from unbiased molecular dynamics simulations. The largest movements involved in the conformational change are the relative movements of the four-helix bundle with respect to the scaffold, which can also be described as movements of the hash-motif with respect to the bundle. Especially, the intracellular parts of TM1 and TM6 undergo large conformational changes. Generally, the presence of both the substrate 5-HT and the ions cause the extracellular cavity to close, hereby stabilizing the extracellular gate interactions decreasing the solvent accessibility of the aromatic lid which seals the substrate site. In addition to a tightening of the extracellular cavity, an opening of the intracellular gate is observed in most of the ten trajectories of 5-HT bound to hSERT (**Sim1–10**). This fits well with the observations made from EPR [36] and FRET [35] studies of LeuT with leucine and sodium ions bound.

**Figure 10:**
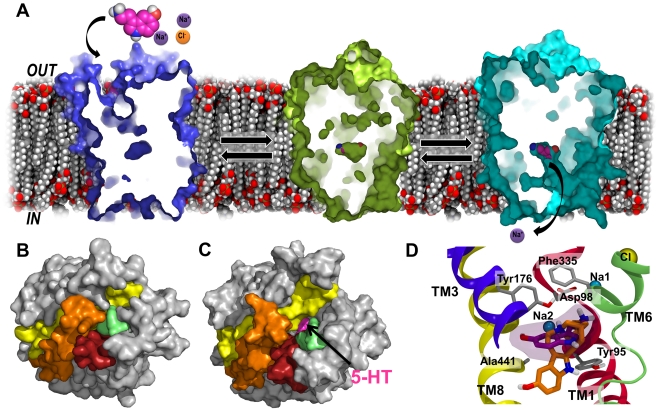
Overview of hSERT conformations and the binding of noribogaine to the inward-facing state. A. The three major conformations and the conformational transitions in 5-HT transport by hSERT is shown with cut-through sections of the transporter viewed from the plane of the membrane bilayer. Left: Outward-facing apo/ions molecular dynamics snapshot (blue) from **Sim26**, Middle: 5-HT/ions homology model in an occluded state (green), Right: Inward-facing conformation of hSERT from **Sim8** at 33.9 ns of simulation (cyan). These three structures reveal the principal states occurring during transport in hSERT. B. Bottom view of the transporter, which is illustrated by surfaces. TM1 (red), TM5 (orange) TM6 (green) and TM8 (yellow) known to line the cytoplasmic pathway have been highlighted together with the ligand in pink. In hSERT homology models which are outward-occluded, no cytoplasmic pathway is found, and the ligand cannot be seen from the cytoplasmic side. C. The snapshot from **Sim8** displaying an inward-facing conformation. A direct connection to the ligand from the intracellular space is found. Only TM1–TM10 are shown here for clarity and comparison. D. Docking pose of noribogaine (cyan) in the inward facing snapshot of hSERT from Sim8 is seen. The ligand is located in the central binding pocket, however, slightly lower than in the validated binding mode of 5-HT (purple) [5].

From the unbiased molecular dynamics simulations of hSERT presented in this study, there is no support to the notion that two substrates are needed for the transporter to assume the inward-facing conformation as has been surmised from binding studies and biased molecular dynamics simulations on LeuT [26] and hDAT [28]. Rather, in hSERT the opposite is observed as the 5-HT molecule, which initially occupies the vestibular S2-site, moves towards the extracellular side. From unbiased molecular dynamics simulation with a single substrate we do indeed observe a transporter with the binding site opening to the intracellular side, see Figure 10A and C. This suggests that hSERT is translocation competent with all three ions and a substrate molecule only in the central S1-binding site. Transport of the sodium ion occupying the Na2-site is observed in addition to early events of substrate transport from additional simulations initiated from the inward-facing transporter lacking the second 5-HT in the S2-site and with no need of additional biasing forces during the simulation. Even though no substrate transport is observed, the transport of the sodium ion from the Na2-site in addition to the great amount of ligand solvation from the intracellular pathway indicates that the transporter is in a fully inward-facing conformation, see Figure 10. The substrate is thus exposed to the intracellular side, but has not moved into the cytoplasm yet. Figure 10C clearly illustrates the cytoplasmic pathway toward the binding pocket holding the substrate, with the substrate visible. In the starting structure of the 5-HT/ions simulation similar substrate binding site exposure is not observed (Figure 10B).

A prediction from the structure of LeuT [8] is that the side chain hydroxyl of Thr354 participates in the coordination of the Na2-ion in addition to several backbone interactions and one additional side chain coordination. From our experimentally validated model of hSERT [3]–[5] the equivalent Asp437 is predicted to be even more important in cation coordination due to the presence of the negatively charged aspartate. From the molecular dynamics simulations it also appears to be the strongest contributor in coordination of the Na2-ion, as it is the last residue to lose the interaction with the sodium ion upon transport.

If binding of a sodium ion to the Na2-site is the governing factor in enabling or driving the conformational changes associated with translocation, then mutation of residues in the Na2-site will have limited consequences for 5-HT transport V_max_ as long as sodium concentrations are saturating. Accordingly, it is expected that the functional affinity of sodium, *i.e.* the K_M_ of Na^+^ in uptake assays, will be severely affected by such a mutation. Indeed, we find that when mutating the Na2-site by the Asp437Asn single-point mutation, the mutant transporter requires considerably higher concentrations of sodium ions to accomplish transport.

We hypothesize that the primary role of the Na1-ion is to facilitate substrate and chloride binding whereas the primary role of the Na2-ion is to drive transport, *i.e.* the rate-limiting step in the translocation cycle would be the transition from the outward-facing conformation to the inward-facing conformation and rapid dissociation of Na2 from the inward-facing conformation would prevent reorientation before 5-HT is released to the cytoplasm. In line with this we find that a compromised sodium affinity for the Na2-site also compromises 5-HT affinity and transport rates. These observations are consistent with the concept that sodium binding to the Na2-site is important for 5-HT binding and that transition from the outward-facing conformation to the inward-facing conformation is dependent on the presence of a sodium ion in the Na2-site.

Release of sodium from the sodium sites into the almost sodium-free intracellular medium constitutes an energetically favorable process that is imperative to transporting 5-HT against a chemical gradient. However, the sodium site tuned to bind sodium in the outward-facing conformation must also release it from the inward-facing conformation. If release of sodium from the Na2-site into the cytoplasm precedes 5-HT release and this process locks the transporter in an inward-facing conformation just after the rate-limiting step in the transport cycle, then we would predict that compromising Na2-ion coordination would result in faster release of Na2 and thus higher transport rates provided extracellular concentrations of sodium are high enough to feed the sodium site. Indeed, the fact that the Asp437Asn mutation significantly impairs apparent sodium affinity while we simultaneously observe no signs of saturation at 150 mM Na^+^ concentration for the Asp437Asn mutant with transport rates comparable to or above wt hSERT implies that if the cells had allowed excess of 150 mM Na^+^ the Asp437Asn mutant would transport much faster than hSERT wt. These observations are consistent with Na2-ion release is the rate-limiting conformational transition preceding 5-HT release from the inward-facing conformation. These data accentuate the important role of the Na2-ion in eliciting the conformational changes that ultimately leads to translocation of the substrate. Our data strongly suggests that the Na2-ion is released prior to release of substrate to the cytoplasm and this release of Na2 is a kinetic latch preventing reorientation back to the extracellular facing conformation without 5-HT translocation.

In the steered molecular dynamics simulations of LeuT [26] and hDAT [28] where substrate translocation is observed, it is noted, that only after deleting the sodium ion at the Na2-site in LeuT, was it possible to observe release of substrate to the cytoplasm using of a biasing pulling force. This is consistent with the hypothesis that release of the Na2-ion is imperative for the inward-facing conformation to stabilize, followed by substrate translocation.

The observations from molecular dynamics simulations and biochemical experiments indicate that Asp437 is the residue contributing most strongly to coordination of the sodium ion occupying the Na2-site. We observe that a conformational change of the χ1 angle of Asp437 initiates the transport of the ion from the Na2 binding site, which is very similar to what has been observed in the molecular dynamics simulations of the LeuT crystal structure by Zhou and Noskov [43]. Similar to what was observed from this study on LeuT, we observe a fast formation of a water wire connecting the Na2 and ligand binding sites to the cytoplasm [43].

Additional evidence supporting the validity of the obtained inward-facing conformation of hSERT is the fact that the non-competitive inhibitor noribogaine [33], which is the active metabolite of ibogaine, can be accommodated in the central substrate binding pocket upon docking studies into the inward-facing conformation of hSERT as revealed in Figure 10D. The main skeleton of noribogaine is similar to 5-HT, and it could therefore be hypothesized that noribogaine is recognized in a same manner as 5-HT, however because of the more bulky three-ring system this ligand is either trapped inside the binding pocket, or it is a very slow substrate. Most other inhibitors of hSERT lock the transporter in an outward-facing conformation, and these inhibitors function by a competitive mechanism [9], [47], [48]. Noribogaine is a non-competitive inhibitor [33], which fits perfectly with it being recognized and partly transported, hereby trapping the transporter in an inward-facing state [33].

We are currently further exploring the effect of a second substrate in the extracellular S2-site in addition to ion dependence of single and double occupied hSERT homology models. It is known that ibogaine induces an inward-facing transporter, and the location of this non-competitive inhibitor is also currently being explored by simulations and biochemically to reveal the inhibitory mechanism. This information is of high importance since knowing the differences in inhibitor mechanisms, will be valuable knowledge in the development of new potential anti-abuse drugs.

## Materials and Methods

### Modeling

All modeling of hSERT in this study is based on a previously experimentally validated homology model of hSERT [3], [5]. Serotonin is placed in the central binding cavity in accordance with experimental results [5], and two sodium ions and one chloride ion are included. Ligands, 5-HT and noribogaine, were prepared for docking as previously described [5]. Docking simulations were carried out using the induced fit protocol [49] in the Schrödinger software packages [50]. A hSERT dimer was used for molecular dynamics simulations built according to the dimeric crystallographic structure of LeuT [8] and the fact that hSERT has been found to be at least a dimer in cell membranes [37], [38] with the interface involving TM12 [39]. In all setups the dimer was inserted into a pre-equilibrated ∼100×130 Å 1-Palmitoyl-2-oleoylphosphatidylcholine (POPC) bilayer by aligning the center of mass of the TM part of the dimer and the center of mass of the POPC patch created by the membrane builder in VMD [51]. The membrane is large enough to provide 15 Å of lipids around the dimer in all directions. The system was solvated with the TIP3P [52] water model and afterwards neutralized with NaCl to a concentration of 200 mM. The sizes of the final systems are ∼100×130×120 Å^3^ yielding approximately 120,000 atoms. All simulations (Table S1) were performed in NAMD 2.6 [53] using periodic boundary conditions and 1 fs time steps. The CHARMM27 force field [54] with CMAP corrections [55], [56]. After minimization, the systems were equilibrated for 2 ns after 500 ps of lipid equilibration. The systems were simulated for either 50 ns (**Sim8a–e** and **Sim11–20**) or 100 ns (all other simulations) including the 2 ns of equilibration and repeated 5 times. The repeats were initiated from the same starting structure. The trajectories were analyzed using the programs VMD and HOLE and 1000 snapshots from each molecular dynamics trajectory. Full details of the modeling can be found in the Supporting Information (please see Text S1).

### Site Specific Mutagenesis

Mutations were introduced by PCR using the Phusion High-Fidelity DNA polymerase (Finnzymes) and primers with appropriate nucleotide mismatches followed by DpnI digestion of the parent DNA. *E.coli* XL10 (Stratagene) were transformed with the mutated DNA and used for DNA production. Mutant constructs were sequenced across the entire reading frame to ensure that no unwanted mutations had been introduced.

### Cell Culture and Expression of hSERT Constructs

The Human Embryonic Kidney cell line HEK-293-MSR was grown and transfected as previously described [5].

### 5-HT Uptake Assays

5-HT uptake kinetics determinations and sodium K_M_'s were measured 40–50 hours after transfection as previously described [5] using a NaCl buffer (150 mM NaCl, K_2_HPO_4_ 1.37 mM, KH_2_PO_4_ 1.37 mM, 0.1 mM CaCl_2_, MgCl_2_ 1 mM, pH 7.4) or NMDG-Cl buffer (150 mM NMDG-Cl, K_2_HPO_4_ 1.37 mM, KH_2_PO_4_ 1.37 mM, 0.1 mM CaCl_2_, MgCl_2_ 1 mM, pH 7.4). NaCl substitution was accomplished with N-methyl-D-glucamine chloride. K_M_'s and V_max_ are the mean from at least three independent experiments.

### Data Calculations

K_m_ and V_max_ were calculated by fitting data to a one site binding hyperbola by nonlinear regression analysis in Graphpad Prism 5.0.

## Supporting Information

Figure S1 A. C_α_ RMSD of transmembrane TM parts of **Sim1–10**. All ten systems reach equilibrium after few nanoseconds of simulation. B. C_α_ RMSF and standard deviations of **Sim1–10** based on alignment on the TM part. As expected the largest movements occur in loop regions, especially in the long extracellular loop 2 between TM3 and TM4, and in the C-terminal part of the protein. C. C_α_ RMSD of transmembrane TM parts of **Sim21–30**. All ten systems reach equilibrium after few nanoseconds of simulation. D. C_α_ RMSF and standard deviations of **Sim21–30** based on alignment on the TM part. As expected the largest movements occur in loop regions, especially in the long extracellular loop 2 between TM3 and TM4, and in the C-terminal part of the protein.
(TIFF)

Figure S2 A. Solvent accessibility surface area (SASA) of the aromatic lid consisting of Tyr176 and Phe335 in **Sim1–10**. Decrease in SASA indicates tightening of the extracellular cavity. B. The hydrogen bond between Tyr350 (TM6) and Glu444 (TM8) is disrupted in more than half of the ten simulations indicating instability of the intracellular gate.
(TIFF)

Figure S3 A. Gate interactions and internal pathway solvation in apo/ions systems. Extracellular gate interactions illustrated by the shortest distance between carboxylate oxygen atoms of Glu493 and guanidinium nitrogen atoms of Arg104. B. Shortest distance between Asp452 carboxylate oxygen atoms and Arg79 guadinium nitrogen atoms in the intracellular gate. C. SASA of the aromatic lid consisting of Tyr176 and Phe335 in **Sim21–30**. Increase in SASA indicates flexibility of the extracellular cavity. D The hydrogen bond between Tyr350 (TM6) and Glu444 (TM8) is disrupted in more than half of the ten simulations indicating instability of the intracellular gate. E. Calculated SASA of the proposed cytoplasmic pathway residues; Phe88, Ser91, Gly94, Gly273, Ser277, Val281, Thr284, Phe347, Ala441, Glu444, and Thr448. F. Number of water molecules located in the extracellular (EC, dark blue, top) and the intracellular cavity (IC, light blue, bottom) in system **Sim26**. Data are extracted from 1000 snapshots during the trajectories.
(TIFF)

Figure S4 The number of water molecules within 3 Å (middle blue), and within 4 Å (light blue) of the cytoplasmic pathway residues Phe88, Ser91, Gly94, Gly273, Ser277, Val281, Thr284, Phe347, Ala441, Glu444, and Thr448 in **Sim8**.
(TIFF)

Figure S5 Coordination of the chloride ion (A) and Na1 (B) in **Sim8**. The interactions remain stable during simulation.
(TIFF)

Figure S6 χ1 dihedral angle (N-CA-CB-CG) of Asp437 in **Sim8**. The jump from approximately 60° to 180° indicates a conformational change from gauche to anti of this side chain. The side chain momentarily jumps to the other gauche conformation around 50 ns.
(TIFF)

Table S1 Overview of trajectories used for analysis.
(DOC)

Text S1 Additional details of methodology used.
(DOC)
