# Supplementary figures and images for: Unbiased Simulations Reveal the Inward-Facing Conformation of the Human Serotonin Transporter and Na+ Ion Release
Source: PLoS Comput Biol. 2011 Oct 27;7(10):e1002246. doi: 10.1371/journal.pcbi.1002246 (PMC3203053; doi:10.1371/journal.pcbi.1002246)

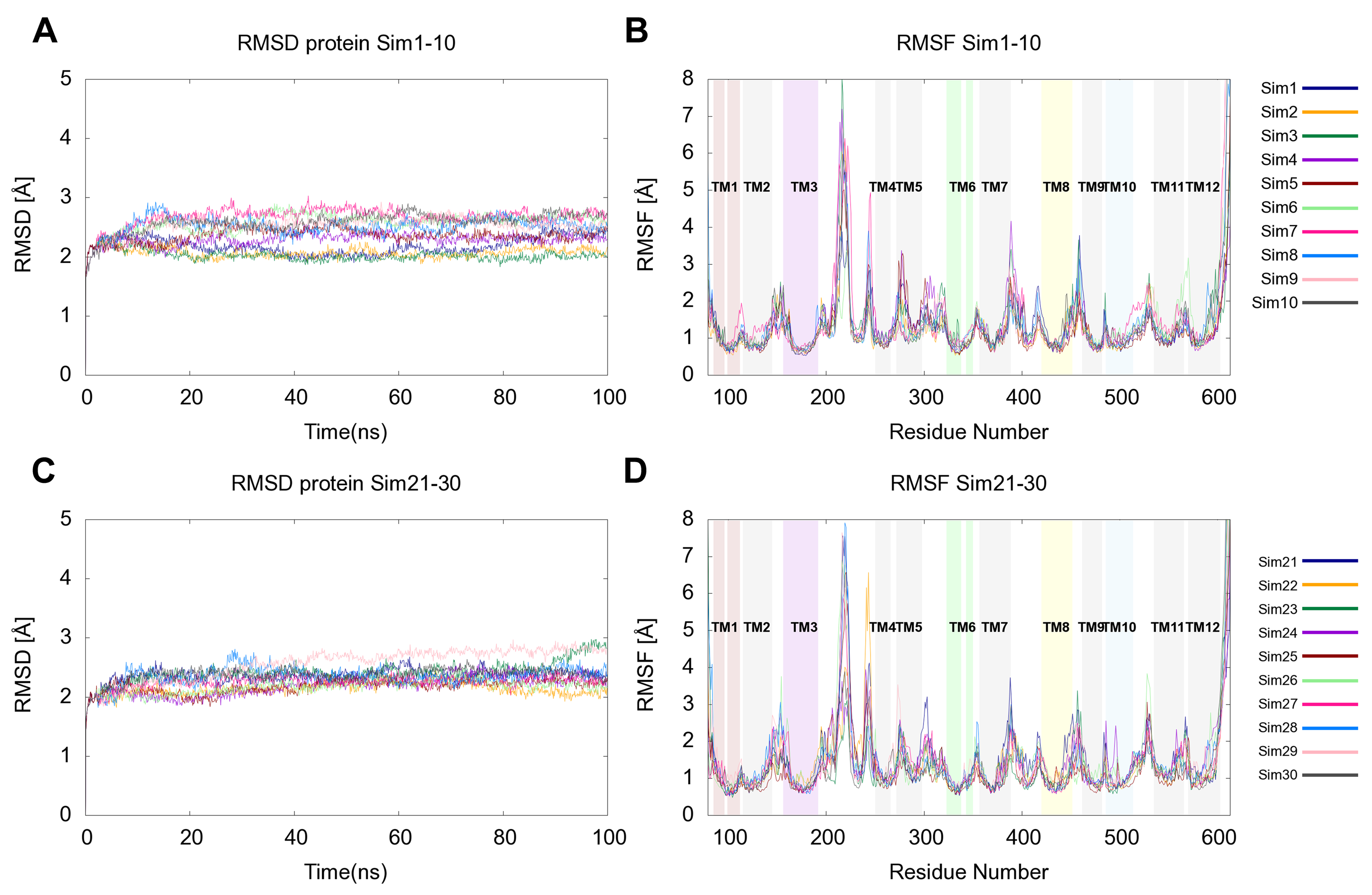

Supplement: Figure S1 — A. Cα RMSD of transmembrane TM parts of Sim1–10. All ten systems reach equilibrium after few nanoseconds of simulation. B. Cα RMSF and standard deviations of Sim1–10 based on alignment on the TM part. As expected the largest movements occur in loop regions, especially in the long extracellular loop 2 between TM3 and TM4, and in the C-terminal part of the protein. C. Cα RMSD of transmembrane TM parts of Sim21–30. All ten systems reach equilibrium after few nanoseconds of simulation. D. Cα RMSF and standard deviations of Sim21–30 based on alignment on the TM part. As expected the largest movements occur in loop regions, especially in the long extracellular loop 2 between TM3 and TM4, and in the C-terminal part of the protein. (TIFF) [file pcbi.1002246.s001.tiff]

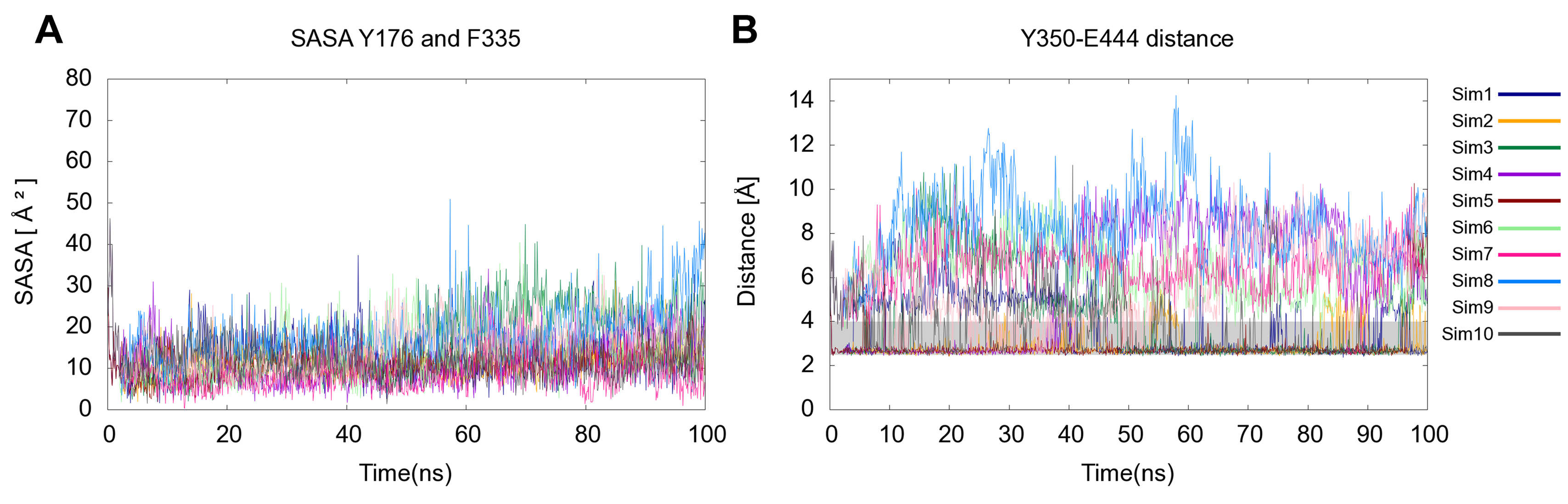

Supplement: Figure S2 — A. Solvent accessibility surface area (SASA) of the aromatic lid consisting of Tyr176 and Phe335 in Sim1–10. Decrease in SASA indicates tightening of the extracellular cavity. B. The hydrogen bond between Tyr350 (TM6) and Glu444 (TM8) is disrupted in more than half of the ten simulations indicating instability of the intracellular gate. (TIFF) [file pcbi.1002246.s002.tiff]

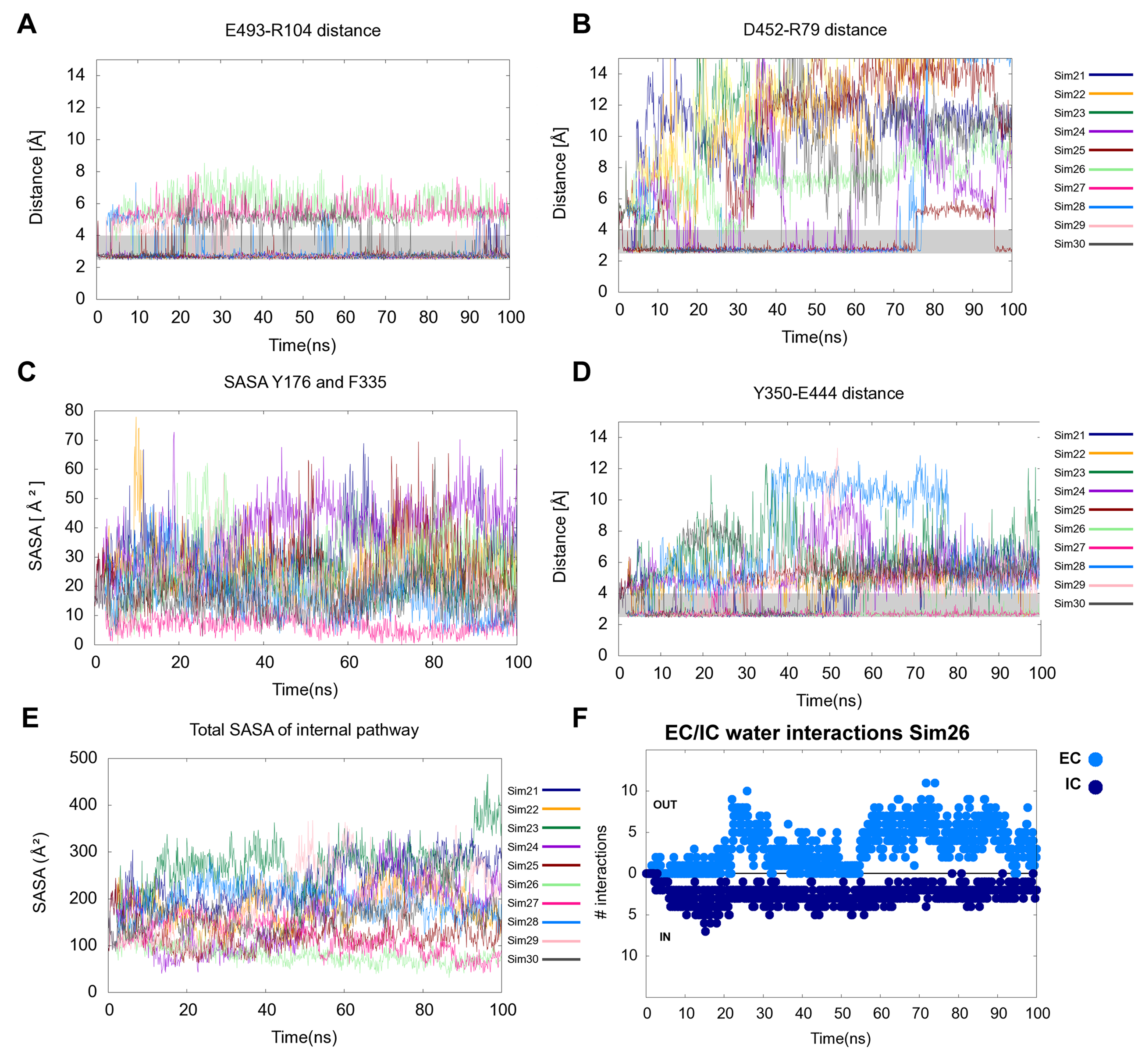

Supplement: Figure S3 — A. Gate interactions and internal pathway solvation in apo/ions systems. Extracellular gate interactions illustrated by the shortest distance between carboxylate oxygen atoms of Glu493 and guanidinium nitrogen atoms of Arg104. B. Shortest distance between Asp452 carboxylate oxygen atoms and Arg79 guadinium nitrogen atoms in the intracellular gate. C. SASA of the aromatic lid consisting of Tyr176 and Phe335 in Sim21–30. Increase in SASA indicates flexibility of the extracellular cavity. D The hydrogen bond between Tyr350 (TM6) and Glu444 (TM8) is disrupted in more than half of the ten simulations indicating instability of the intracellular gate. E. Calculated SASA of the proposed cytoplasmic pathway residues; Phe88, Ser91, Gly94, Gly273, Ser277, Val281, Thr284, Phe347, Ala441, Glu444, and Thr448. F. Number of water molecules located in the extracellular (EC, dark blue, top) and the intracellular cavity (IC, light blue, bottom) in system Sim26. Data are extracted from 1000 snapshots during the trajectories. (TIFF) [file pcbi.1002246.s003.tiff]

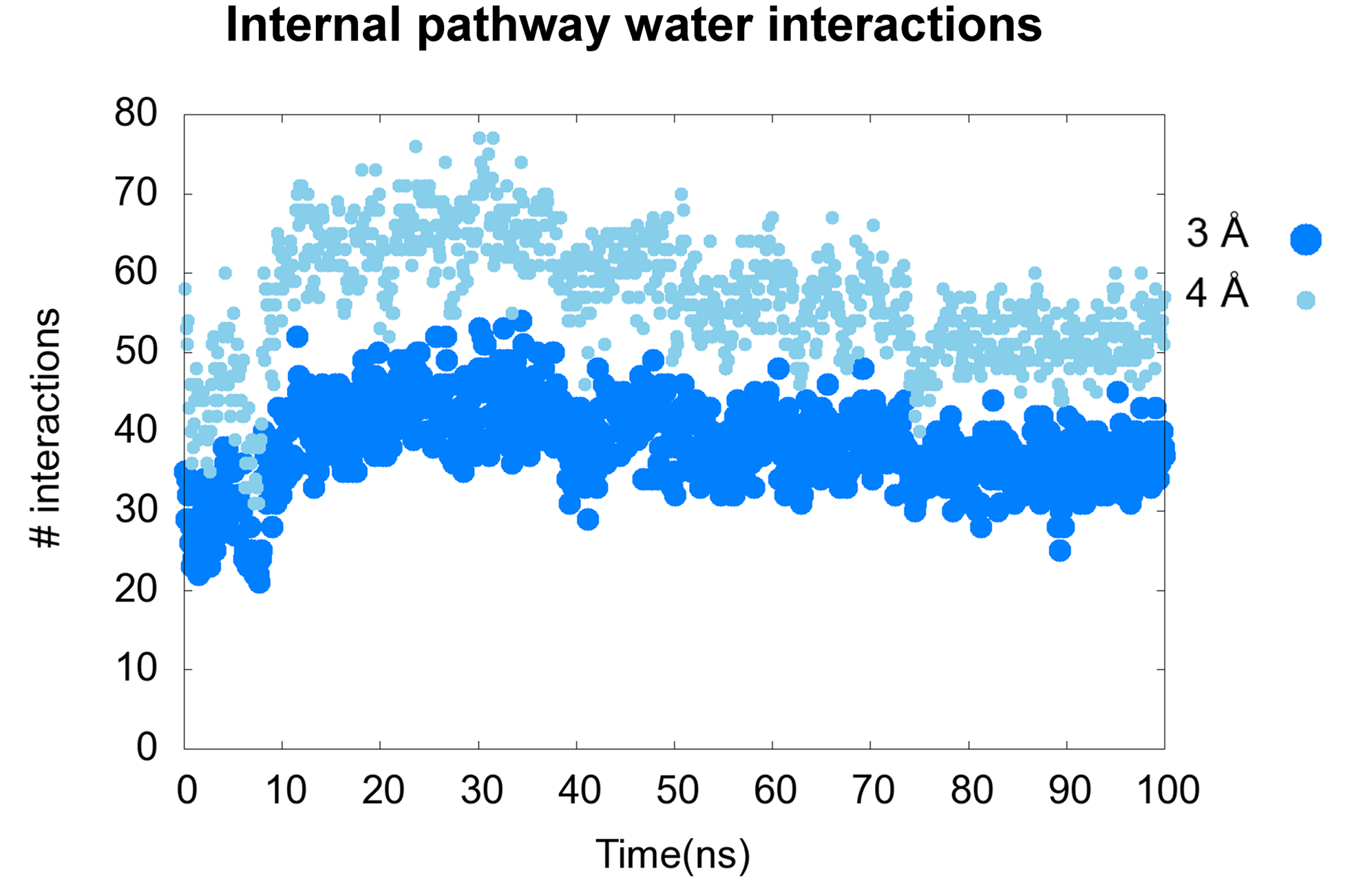

Supplement: Figure S4 — The number of water molecules within 3 Å (middle blue), and within 4 Å (light blue) of the cytoplasmic pathway residues Phe88, Ser91, Gly94, Gly273, Ser277, Val281, Thr284, Phe347, Ala441, Glu444, and Thr448 in Sim8. (TIFF) [file pcbi.1002246.s004.tiff]

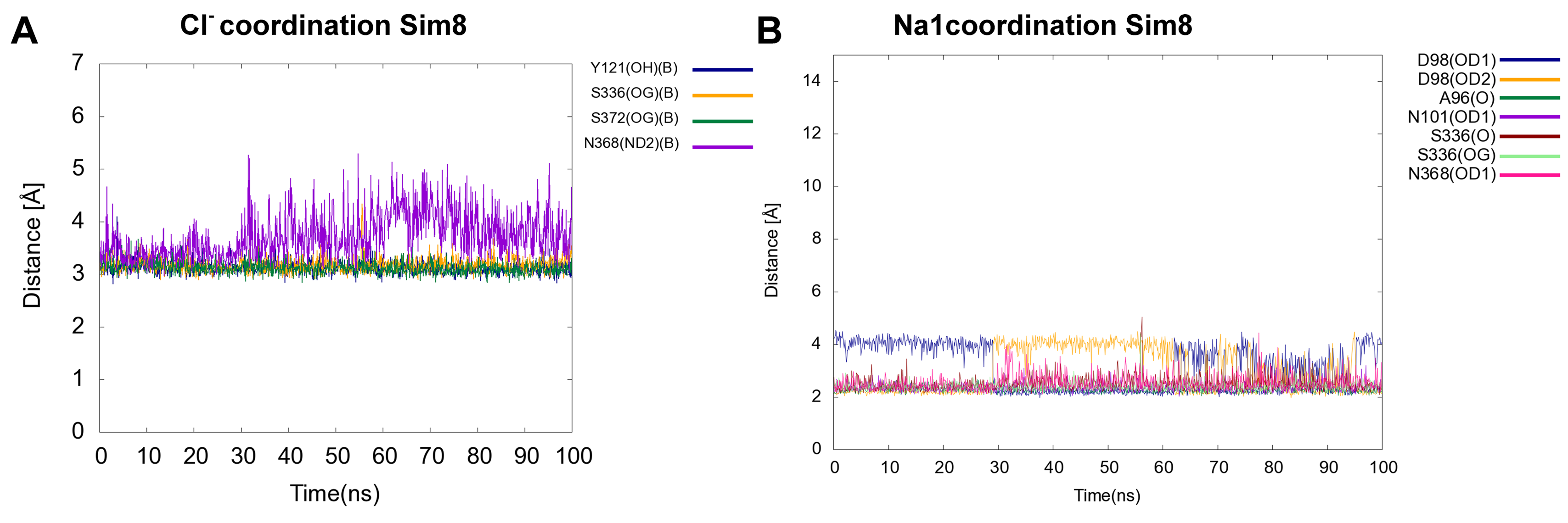

Supplement: Figure S5 — Coordination of the chloride ion (A) and Na1 (B) in Sim8. The interactions remain stable during simulation. (TIFF) [file pcbi.1002246.s005.tiff]

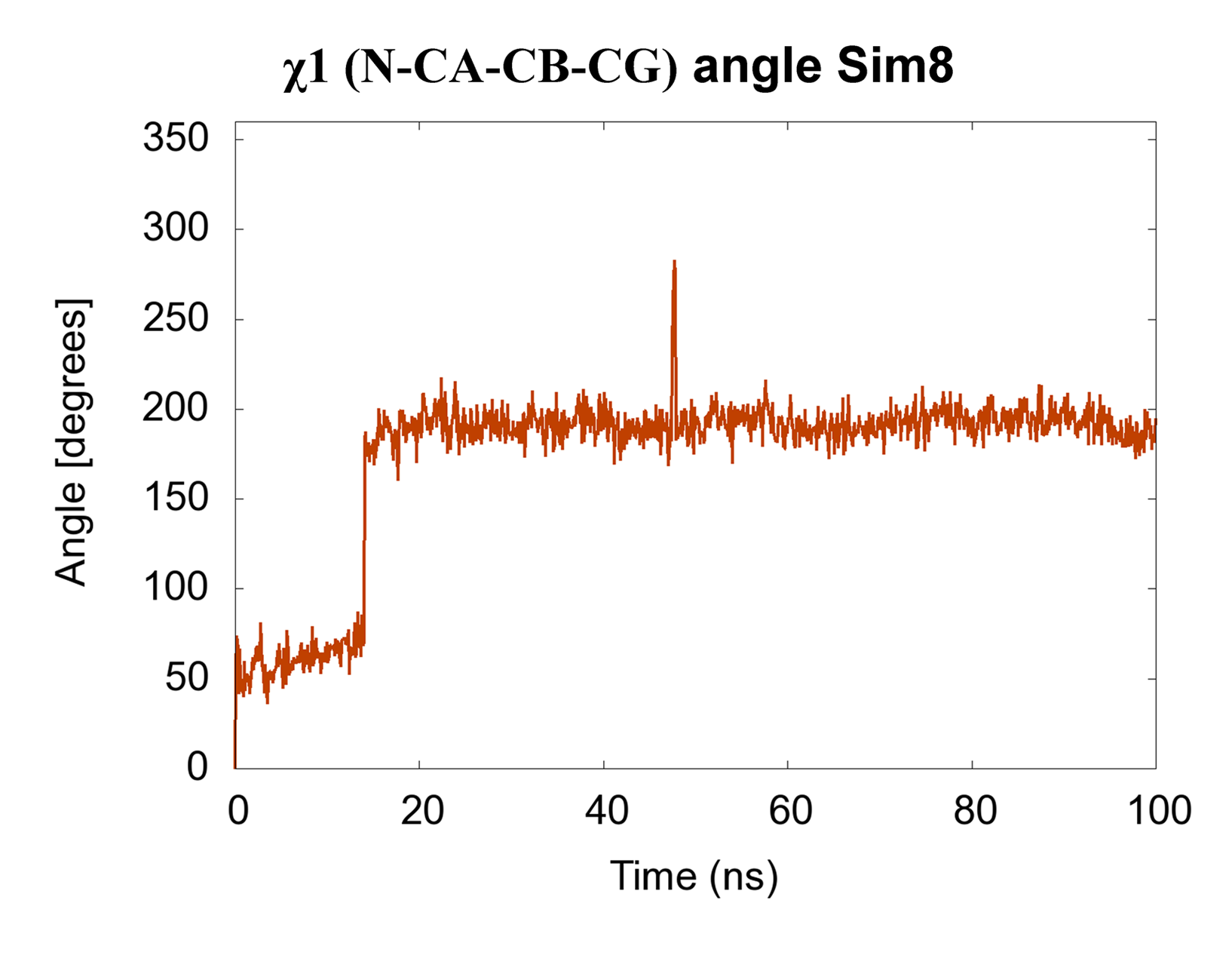

Supplement: Figure S6 — χ1 dihedral angle (N-CA-CB-CG) of Asp437 in Sim8. The jump from approximately 60° to 180° indicates a conformational change from gauche to anti of this side chain. The side chain momentarily jumps to the other gauche conformation around 50 ns. (TIFF) [file pcbi.1002246.s006.tiff]
