# Supplementary material for: Unbiased Simulations Reveal the Inward-Facing Conformation of the Human Serotonin Transporter and Na+ Ion Release
Source: PLoS Comput Biol. 2011 Oct 27;7(10):e1002246. doi: 10.1371/journal.pcbi.1002246 (PMC3203053; doi:10.1371/journal.pcbi.1002246)
Supplement: Table S1 — Overview of trajectories used for analysis. (DOC) [file pcbi.1002246.s007.doc]

**Table S1.** Overview of hSERT systems used for analysis. hSERT is simulated as a dimer giving two trajectories for each MD simulation, resulting in 10 trajectories from the five repeats. The name of each system for analysis is listed in column 1 or 2. S1-site and S2-site columns indicate what is present in this site. The simulation times are listed in the Time-column. All systems contain the two sodium ions (Na1 and Na2) and the chloride ion.

| **Trajectory** | **Prolonged**  **Trajectory** | **S1-site** | **S2-site** | **Time** |
| --- | --- | --- | --- | --- |
| **Sim1** |  | 5-HT | None | 100 ns |
| **Sim2** |  | 5-HT | None | 100 ns |
| **Sim3** |  | 5-HT | None | 100 ns |
| **Sim4** |  | 5-HT | None | 100 ns |
| **Sim5** |  | 5-HT | None | 100 ns |
| **Sim6** |  | 5-HT | None | 100 ns |
| **Sim7** |  | 5-HT | None | 100 ns |
| **Sim8** |  | 5-HT | None | 100 ns |
|  | **Sim8a** | 5-HT | None | 50 ns |
|  | **Sim8b** | 5-HT | None | 50 ns |
|  | **Sim8c** | 5-HT | None | 50 ns |
|  | **Sim8d** | 5-HT | None | 50 ns |
|  | **Sim8e** | 5-HT | None | 50 ns |
| **Sim9** |  | 5-HT | None | 100 ns |
| **Sim10** |  | 5-HT | None | 100 ns |
| **Sim11** |  | 5-HT | 5-HT | 50 ns |
| **Sim12** |  | 5-HT | 5-HT | 50 ns |
| **Sim13** |  | 5-HT | 5-HT | 50 ns |
| **Sim14** |  | 5-HT | 5-HT | 50 ns |
| **Sim15** |  | 5-HT | 5-HT | 50 ns |
| **Sim16** |  | 5-HT | 5-HT | 50 ns |
| **Sim17** |  | 5-HT | 5-HT | 50 ns |
| **Sim18** |  | 5-HT | 5-HT | 50 ns |
| **Sim19** |  | 5-HT | 5-HT | 50 ns |
| **Sim20** |  | 5-HT | 5-HT | 50 ns |
| **Sim21** |  | None | None | 100 ns |
| **Sim22** |  | None | None | 100 ns |
| **Sim23** |  | None | None | 100 ns |
| **Sim24** |  | None | None | 100 ns |
| **Sim25** |  | None | None | 100 ns |
| **Sim26** |  | None | None | 100 ns |
| **Sim27** |  | None | None | 100 ns |
| **Sim28** |  | None | None | 100 ns |
| **Sim29** |  | None | None | 100 ns |
| **Sim30** |  | None | None | 100 ns |
