# Supplementary material for: Unbiased Simulations Reveal the Inward-Facing Conformation of the Human Serotonin Transporter and Na+ Ion Release
Source: PLoS Comput Biol. 2011 Oct 27;7(10):e1002246. doi: 10.1371/journal.pcbi.1002246 (PMC3203053; doi:10.1371/journal.pcbi.1002246)
Supplement: Text S1 — Additional details of methodology used. (DOC) [file pcbi.1002246.s008.doc]

**Text S1**

**Homology Modeling:** A homology model of hSERT based on the crystal structure of the bacterial homologue LeuT*_Aa_* [1] has been used in this study. The alignment applied in the model building is the extensively refined one of neurotransmitter sodium symporters described by Weinstein and co-workers [2]. The model has been validated in Celik *et al.* [3] and the extracellular loop 2 connecting TM3 and TM4 has been further optimized as described in Koldsø *et al.* [4]. The two sodium ions have been included in the same positions as in Yamashita *et al.* [1]. The chloride ion has been incorporated in the proposed binding site [5,6] followed by a brief minimization.

**Ligand Modeling:** 5-HT was prepared for IFD as described in Celik *et al.* [3] and noribogaine was prepared in a similar way: Noribogaine was built in Maestro [7] with the tertiary amine modeled as charged in accordance with a pK_a_ value of 10.13 predicted by Epik 2.1 [8] (Schrödinger Suite 2010). Noribogaine was minimized in implicit solvent using the conjugate gradient method in MacroModel 9.8 with a maximum number of iterations of 50,000 or until convergence [9] The minimized structure served as input for a Low-Mode Conformational Search [10]. The OPLS-AA force field [11-13] was used both in the minimization and conformational search. The lowest energy structure was chosen as input for the IFD calculations.

**Protein Preparation (for Induced Fit Docking):** The homology model was prepared for induced fit docking (IFD) of 5-HT in the S1-site utilizing the protein preparation wizard in the Schrödinger 2008 suite (Schrödinger LLC) For the S2-site dockings the MD structure at 0 ns was used, whereas the 33.9 ns snapshot of **Sim8** was used for noribogaine docking. Both structures were minimized for 15,000 steps of conjugate gradient in NAMD [14] followed by protein preparation in Schrödinger 2010 [15] prior to IFD.

**Protein Preparation (for MD simulations):** hSERT is simulated as a dimer based on the dimer structure observed in the LeuT*_Aa_* crystal structure [1]. This dimer structure has previously been applied in simulations of LeuT*_Aa_* [16,17] and has been found for hSERT in cell membranes [18,19]. The three acidic residues Glu453, Glu508 and Asp524 have been modeled as protonated during the simulations on the basis of pK_a_ predictions made by PROPKA2.0 [20]. Glu508 contribute to a Glu-Glu pair with Glu136 just below the central binding pocket. The analogous position of Glu508 (Glu419) in the LeuT*_Aa_* crystal structure has similarly been proposed to be protonated because of it is involved in a similar Glu-Glu pair with Glu62 (LeuT) [5]. Two of the histidine residues have been modeled as histidinium, namely His223 and His240. The rest of the histidines (His143, His235, His456) are N1δ protonated according to PROPKA results and visual inspection. The PSFGEN plug-in for VMD [21] using the CHARMM27 topology files have been used to determine hydrogen positions and include the disulfide bridge between Cys200–Cys209. In all systems the dimer has been inserted into a pre-equilibrated ~100×130 Å POPC bilayer by aligning the center of mass of the transmembrane part of the dimer and the center of mass of the POPC patch created by the membrane builder in VMD [21]. The membrane is large enough to provide 15 Å of lipids around the dimer in all directions. The system was solvated and afterwards neutralized with NaCl to a physiological concentration of 200 mM using the SOLVATE and AUTOIONIZE plug-ins of VMD [21]. The sizes of the final systems are ~ 100×130×120 Å^3^ yielding approximately 120,000 atoms.

**Induced fit docking (IFD):** 5-HT has been docked into the central S1-site by the IFD [22-24] as previously described [3]. IFDs of 5-HT into the S2-site and noribogaine into S1-site of hSERT (**Sim8** 33.9 ns snapshot) were done in Schrödinger 2010 suite [25-27]. The binding site in IFD of 5-HT in S2-site was defined from three residues; Arg104, Phe407 and Glu493; as the corresponding residues in LeuT (Arg30, Phe324 and Asp404) enclose the extracellular cavity binding site [28]. In the IFD of noribogaine to the **Sim8** snapshot at 33.9 ns, the binding site was defined by 5-HT. The Standard Precision (SP) scoring function [29] and Extra Precision (XP) scoring function [30] were used in initial and redocking stage respectively.

**Simulation protocol:** All simulations are performed in NAMD2.6 [14] using periodic boundary conditions and 1 fs time steps. The CHARMM27 force field [31] with CMAP corrections [32,33] and the TIP3P water model [34] for explicit solvent have been applied. The complete systems (**Sim1-30**) were minimized with conjugated gradient method for 1,500 steps. The minimization was followed by a 500 ps NVT lipid tail melting at 310 K where everything except the lipid tails are held fixed. The prolonged simulations (**Sim8a-e**) were minimized for 5,000 steps of conjugated gradient prior to simulation. The system was equilibrated in a 2 ns constraint free simulation in the NPT ensemble at 1 atm pressure and 310 K. Van der Waals interactions were calculated applying a cut-off distance of 12 Å and long range electrostatics are calculated utilizing the Particle Mesh Ewald (PME) algorithm [35]. Constant pressure of 1 atm was maintained by the Langevin piston method [36] with a piston period of 100 fs and a piston decay of 50 fs. Constant temperature of 310 K is achieved utilizing the Langevin dynamics with a damping constant of 1 ps^-1^ in lipid melting simulation and 5 ps^-1^ in the equilibration phase. The systems were simulated either for 50 ns (**Sim8a-e** and **Sim11-20**) or 100 ns (**Sim1-10** and **Sim21-30**) including the 2 ns of equilibration and repeated 5 times from the same starting structure. The differences observed in the simulations evolve from the MD simulation protocol. For complete system setup see table S1, where **Sim1-5**, **Sim11-15**, **Sim21-25** originates from monomer A, and **Sim6-10**, **Sim8a-e**, **Sim16-20**, and **Sim25-30** originates from monomer B.

**MD Analysis:** Solvent accessible surface area (SASA), root-mean-square deviations (RMSD) and fluctuations (RMSF) of Cα atoms have been calculated employing build-in functions in VMD [21], whereas the pore radius was calculated using the program HOLE [37,38]. For the analysis 1000 structures from the trajectories were used.

**SI references**

1. Yamashita A, Singh SK, Kawate T, Jin Y, Gouaux E. (2005) Crystal structure of a bacterial homologue of Na^+^/Cl^-^-dependent neurotransmitter transporters. Nature 437: 215-223.

2. Beuming T, Shi L, Javitch JA, Weinstein H. (2006) A comprehensive structure-based alignment of prokaryotic and eukaryotic Neurotransmitter/Na^+^ symporters (NSS) aids in the use of the LeuT structure to probe NSS structure and function. Mol Pharmacol 70: 1630-1642.

3. Celik L, Sinning S, Severinsen K, Hansen C, Møller M, et al. (2008) Binding of serotonin to the human serotonin transporter. molecular modeling and experimental validation. J Am Chem Soc 130: 3853-3865.

4. Koldsø H, Severinsen K, Tran TT, Celik L, Jensen HH, et al. (2010) The two enantiomers of citalopram bind to the human serotonin transporter in reversed orientations. J Am Chem Soc 132: 1311-1322.

5. Forrest LR, Tavoulari S, Zhang YW, Rudnick G, Honig B. (2007) Identification of a chloride ion binding site in Na^+^/Cl^-^-dependent transporters. Proc Natl Acad Sci USA 104: 12761-12766.

6. Zomot E, Bendahan A, Quick M, Zhao Y, Javitch J, et al. (2007) Mechanism of chloride interaction with neurotransmitter:Sodium symporters. Nature 449: 726-730.

7. Maestro, version 9.1, Schrödinger, LLC, New York, NY, 2010.

8. Epik, version 2.1, Schrödinger, LLC, New York, NY, 2010.

9. MacroModel, version 9.8, Schrödinger, LLC, New York, NY, 2010.

10. Kolossváry I, Guida WC. (1996) Low mode search. an efficient, automated computational method for conformational analysis: Application to cyclic and acyclic alkanes and cyclic peptides. J Am Chem Soc 118: 5011-5019.

11. Jorgensen WL, Tirado-Rives J. (1988) The OPLS potential functions for proteins - energyminimizations for crystals of cyclic-peptides and crambin. J Am Chem Soc 110: 1666-1671.

12. Jorgensen WL, Maxwell DS, Tirado-Rives J. (1996) Development and testing of the OPLS all-atom force field on conformational energetics and properties of organic liquids. J Am Chem 118: 11225-11236.

13. Kaminski GA, Friesner RA, Tirado-Rives J, Jorgensen WL. (2001) Evaluation and reparametrization of the OPLS-AA force field for proteins via comparison with accurate quantum chemical calculations on peptides. J Phys Chem B 105: 6474-6487.

14. Phillips JC, Braun R, Wang W, Gumbart J, Tajkhorshid E, et al. (2005) Scalable molecular dynamics with NAMD. J Comput Chem 26: 1781-1802.

15. Schrödinger Suite 2010 Protein Preparation Wizard, Epik version 2.1, Schrödinger LLC, New York, NY, 2010, Impact version 5.6, Schrödinger, LLC, New York, NY, 2010, Prime version 2.2, Schrödinger, LLC, New York, NY, 2010.

16. Celik L, Schiøtt B, Tajkhorshid E. (2008) Substrate binding and formation of an occluded state in the leucine transporter. Biophys J 94: 1600-1612.

17. Shaikh SA, Tajkhorshid E. (2010) Modeling and dynamics of the inward-facing state of a Na+/Cl- dependent neurotransmitter transporter homologue. PLoS Comput Biol 6: e1000905. doi:10.1371/journal.pcbi.1000905

18. Sitte HH, Farhan H, Javitch JA. (2004) Sodium-dependent neurotransmitter transporters: Oligomerization as a determinant of transporter function and trafficking. Mol Interv 4: 38-47.

19. Fjorback AW, Pla P, Müller HK, Wiborg O, Saudou F, et al. (2009) Serotonin transporter oligomerization documented in RN46A cells and neurons by sensitized acceptor emission FRET and fluorescence lifetime imaging microscopy. Biochem Biophys Res Commun 380: 724-728.

20. Li H, Robertson AD, Jensen JH. (2005) Very fast empirical prediction and rationalization of protein pK_a_ values. Proteins: Struct Funct Bioinf 61: 704-721.

21. Humphrey W, Dalke A, Schulten K. (1996) VMD: Visual molecular dynamics. J Mol Graph 14: 33-38.

22. Schrödinger Suite 2008 Induced Fit Docking protocol, Glide version 5.0, Schrödinger, LLC, New York, NY, 2005, Prime version 1.7, Schrödinger, LLC, New York, NY, 2005. .

23. Glide, version 5.0, Schrödinger, LLC, New York, NY, 2008.

24. Prime, version 2.0, Schrödinger, LLC, New York, NY, 2008.

25. Glide, version 5.6, Schrödinger, LLC, New York, NY, 2010.

26. Prime, version 2.2, Schrödinger, LLC, New York, NY, 2010.

27. Schrödinger Suite 2010 Induced Fit Docking protocol, Glide version 5.6, Schrödinger, LLC, New York, NY, 2010, Prime version 2.2, Schrödinger, LLC, New York, NY, 2010.

28. Shi L, Quick M, Zhao Y, Weinstein H, Javitch JA. (2008) The mechanism of a neurotransmitter:sodium symporter—Inward release of Na+ and substrate is triggered by substrate in a second binding site. Mol Cell 30: 667-677.

29. Friesner RA, Banks JL, Murphy RB, Halgren TA, Klicic JJ, et al. (2004) Glide: A new approach for rapid, accurate docking and scoring. 1. method and assessment of docking accuracy. J Med Chem 47: 1739-1749.

30. Friesner RA, Murphy RB, Repasky MP, Frye LL, Greenwood JR, et al. (2006) Extra precision glide: Docking and scoring incorporating a model of hydrophobic enclosure for Protein-Ligand complexes. J Med Chem 49: 6177-6196.

31. MacKerell ADJ, Bashford D, Bellott M, Dunbrack Jr. RL, Evanseck JD, et al. (1998) All-atom empirical potential for molecular modeling and dynamics studies of proteins. J Phys Chem B 102: 3586-3616.

32. MacKerell ADJ, Feig M, Brooks CL III. (2004) Extending the treatment of backbone energetics in protein force fields: Limitations of gas-phase quantum mechanics in reproducing protein conformational distributions in molecular dynamics simulations. J Comput Chem 25: 1400-1415.

33. MacKerell ADJ, Feig M, Brooks CL III. (2004) Improved treatment of the protein backbone in empirical force fields. J Am Chem Soc 126: 698-699.

34. Jorgensen WL, Chandrasekhar J, Madura JD, Impey RW, Klein ML. (1983) Comparison of simple potential functions for simulating liquid water. J Chem Phys 79: 926-935.

35. Darden T, York D, Pedersen L. (1993) Particle mesh ewald: An *N* *log(*N)* method for ewald sums in large systems. J Chem Phys 98: 10089-10092.

36. Feller SE, Zhang Y, Pastor RW, Brooks BR. (1995) Constant pressure molecular dynamics simulation: The langevin piston method. J Chem Phys 103: 4613-4621.

37. Smart OS, Goodfellow JM, Wallace BA. (1993) The pore dimensions of gramicidin A. Biophys J 65: 2455-2460.

38. Smart OS, Neduvelil JG, Wang X, Wallace BA, Sansom MSP. (1996) HOLE: A program for the analysis of the pore dimensions of ion channel structural models. J Mol Graph 14: 354-360.
